# Development and verification of a physiologically motivated internal controller for the open-source extended Hill-type muscle model in LS-DYNA

**DOI:** 10.1007/s10237-023-01748-9

**Published:** 2023-08-05

**Authors:** Oleksandr V. Martynenko, Fabian Kempter, Christian Kleinbach, Lennart V. Nölle, Patrick Lerge, Syn Schmitt, Jörg Fehr

**Affiliations:** 1 Institute for Modelling and Simulation of Biomechanical Systems, University of Stuttgart, Nobelstr. 15, 70569 Stuttgart, Germany; 2 Institute of Engineering and Computational Mechanics, University of Stuttgart, Pfaffenwaldring 9, 70569 Stuttgart, Germany

**Keywords:** Hill-type muscle model, Finite element analysis, Human body model, Muscle control, Active muscle modelling, LS-DYNA

## Abstract

Nowadays, active human body models are becoming essential tools for the development of integrated occupant safety systems. However, their broad application in industry and research is limited due to the complexity of incorporated muscle controllers, the long simulation runtime, and the non-regular use of physiological motor control approaches. The purpose of this study is to address the challenges in all indicated directions by implementing a muscle controller with several physiologically inspired control strategies into an open-source extended Hill-type muscle model formulated as LS-DYNA user-defined umat41 subroutine written in the Fortran programming language. This results in increased usability, runtime performance and physiological accuracy compared to the standard muscle material existing in LS-DYNA. The proposed controller code is verified with extensive experimental data that include findings for arm muscles, the cervical spine region, and the whole body. Selected verification experiments cover three different muscle activation situations: (1) passive state, (2) open-loop and closed-loop muscle activation, and (3) reflexive behaviour. Two whole body finite element models, the 50th percentile female VIVA OpenHBM and the 50th percentile male THUMS v5, are used for simulations, complemented by the simplified arm model extracted from the 50th percentile male THUMS v3. The obtained results are evaluated additionally with the CORrelation and Analysis methodology and the mean squared error method, showing good to excellent biofidelity and sufficient agreement with the experimental data. It was shown additionally how the integrated controller allows simplified mimicking of the movements for similar musculoskeletal models using the parameters transfer method. Furthermore, the Hill-type muscle model presented in this paper shows better kinematic behaviour even in the passive case compared to the existing one in LS-DYNA due to its improved damping and elastic properties. These findings provide a solid evidence base motivating the application of the enhanced muscle material with the internal controller in future studies with Active Human Body Models under different loading conditions.

**Supplementary Information:**

The online version contains supplementary material available at 10.1007/s10237-023-01748-9.

## Introduction

**Fig. 1:**
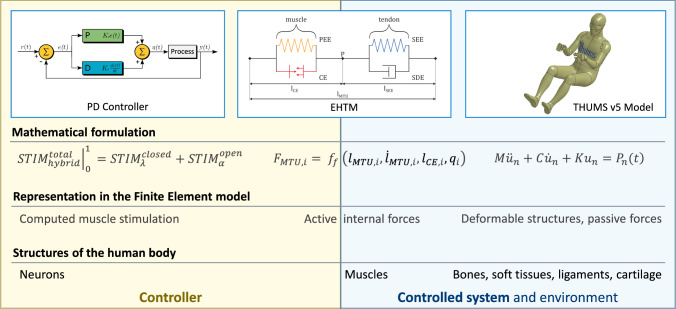
Typical logical structure of the finite element active human body model (AHBM)

The constantly growing number of vehicles being partially or highly automated requires a change in their research and development paradigm due to the implementation of advanced safety systems, which will ensure integrated safety in the future. More and more virtual modelling and testing procedures utilising different numerical methods are used in practice to cope with this need. Computational Human Body Models (HBMs) are considered an adequate representation of the vehicle occupants during these procedures (van Ratingen 2016; Östling et al 2019). To satisfy the increasing demands for vehicle safety systems development, HBMs must replicate the behaviour of living humans as closely as possible. This is the case not only for high-speed impacts and in-crash scenarios but also for low-speed collisions and pre-crash manoeuvres. For these purposes, HBMs are enhanced by additional model parts representing muscles and complementary software code that controls muscle recruitment, becoming Active Human Body Models (AHBMs). The typical structure of such a model, the muscle material, and the control approach used in the current study are shown in Fig. 1. AHBMs are ideal surrogate models for the scenarios characterized by dynamic occupant behaviour as they are much more compliant due to their material properties derived from experiments with living humans (Iwamoto and Nakahira 2015; Shelat et al 2016; Devane et al 2019). Moreover, supplementary muscle controllers allow for the generation of nervous signals during runtime resulting in the ability to simulate involuntary or voluntary reflexes and purposeful protective or evasive movements, therefore being essential for enhancing the occupant safety of future vehicles.

Researchers have shown an increased interest in developing AHBMs with controllers and active muscle elements included in the whole body in recent years. The Total HUman Model for Safety v5 (THUMS v5) model incorporates Proportional Integral Derivative (PID) closed-loop controllers operating on the angles between 17 body parts (Posture controller) and contact forces between limbs and vehicle environment (Contact force controller) (Iwamoto and Nakahira 2015; Kato et al 2017). Based on the PID controller’s output, the activation level for each muscle is calculated using additional numerical transformations like muscle activation coefficients, the sigmoid function for a transition from stimulation to activation based on the firing rate of motor neurons and muscle percentage contributions to the motion in a specific joint due to muscles redundancy. The Global Human Body Models Consortium (GHBMC) M50-OS + Active model utilizes the same controller strategy as THUMS v5 but with differently tuned PID control parameters, based on diverse validation and model data (Devane et al 2019). It is worthwhile to mention that the GHBMC model authors stated that using PID controllers in the AHBM could only be considered as a simplified mathematical control model not reflecting the true complexity of the neuromuscular control system (Devane et al 2019). Therefore, they proposed an additional closed-loop controller for neck muscle activation based on the natural reflex mechanisms in a latter study (Correia et al 2021). Two PID controllers representing the vestibulocollic reflex (VCR) and cervicocollic reflex (CCR) were implemented and validated with experimental data, wherein the CCR controller signal depends on the muscle stretch as input. A similar approach for the neck muscles is taken in the VIVA OpenHBM model, which has implemented angular position feedback (APF) and muscle length feedback (MLF) controllers with optimised gains but no additional controllers for other body regions (Putra et al 2020). The SAFER HBM (Östh et al 2015), in addition to the full-body omni-directional APF and MLF closed-loop controllers (Ólafsdóttir et al 2019), has a dedicated shoulder muscle feedback controller to model the load transfer from the steering wheel to the torso, which was added recently (Fice et al 2021). The model uses PID controllers to govern the angles between body parts and selected muscle lengths in the neck and upper extremity regions. In addition, the distribution of muscle activation levels was evaluated based on in vivo data retrieved from experiments. A set of two first-order differential equations is used to transfer muscle stimulation levels to activation emulating muscle activation dynamics (Ólafsdóttir et al 2019). Two other proprietary models with full body muscle control functions, the THUMS TUC-VW AHBM (Yigit 2018) and the A-THUMS-D (Martynenko et al 2019; Mishra et al 2020), use MLF controller and physiologically motivated activation dynamics to model the occupant behaviour during evasive manoeuvres. The THUMS TUC-VW AHBM is intended for use in the Virtual Performance Solution (VPS) software (ESI Group, Rungis, France) in contrast to all other models that run in LS-DYNA (LSTC, Livermore, CA, US).

**Fig. 2:**
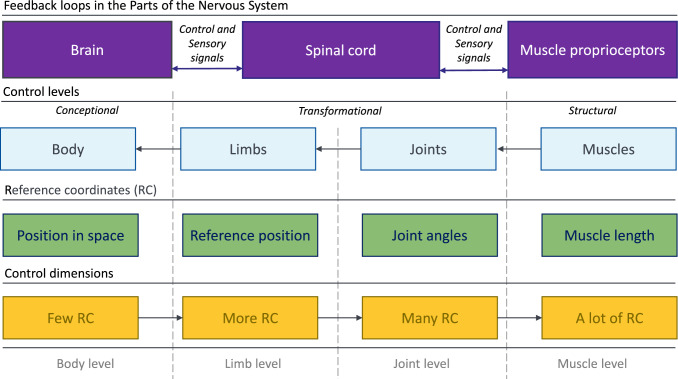
Layered hierarchical architecture of the human motor control, which represents two internal processes: (1) few-to-many mapping from the low-dimensional reference coordinates (RC) on the body level to multidimensional RC on the muscle level (control dimensions); (2) back-coupling feedback loops from the brain to muscles level and vice-versa both within the central nervous system and through different receptors inside the body (Latash 2021; Walter et al 2021)

The implementation of the APF muscle control strategy with LS-DYNA keywords typically involves the following steps: (1) insertion of 1D truss finite elements with the *MAT_MUSCLE material (alternate name *MAT_156 material) for muscle representation; (2) definition of the desired angles between the model’s body parts to control; (3) application of the built-in *PIDCTL function for error signal calculation; and (4) coding of different mathematical functions for extensive controller related calculations (LSTC 2016). In the case of MLF, the procedure becomes even more complicated since the length of almost every muscle string (element or number of elements) needs to be controlled with a separate PID controller or additional code, which redistributes muscle activation signals depending on the muscle percentage contribution based on in vivo experimental data. All these steps are done in the keyword modelling language of LS-DYNA, which adds a large amount of computational load to the already resource-demanding simulations with HBMs. In addition, the latest motor control studies suggest that humans plan their movements using a layered hierarchical control architecture (Latash 2021; Walter et al 2021) which is schematically shown in Fig. 2. Such a hypothesis additionally implies that in the best case the controller structure for AHMB should be designed in several layers: a *‘conceptional’* where the movement is planned, a *‘structural’* where the muscles stimulation signal is generated, and several intermediate *‘transformational’* layers where the muscle-joint redundancy is resolved (Walter et al 2021). Herewith, the *‘transformational’* layers operate on the whole physiological set of joints of the body affecting muscles, but not on the artificially defined angles between body parts of the model. As a solution, we propose using MLF control through implementing a computationally effective and physiologically motivated controller on the lowest possible hierarchical layer inside a muscle material for the truss finite element (FE) in LS-DYNA. Due to the direct access to the LS-DYNA internal variables, such a controller is able to function independently and govern the reflexes enabling independent posture maintenance or rudimental reactions to the acceleration vector change. Moreover, it will allow AHBMs to simulate bracing for impact or steering manoeuvres when supplemented by an interface to receive additional control signals from the required number of overlying control layers.

Consequently, we postulate the following research objectives for our manuscript: to extend the recently published open-source extended Hill-type muscle (EHTM) material model for LS-DYNA (Kleinbach et al 2017) by efficient physiological control approaches; to accelerate AHBM simulations in LS-DYNA by freely available controller code implementation inside a user-defined material (Nölle et al 2022); to verify this implementation with the existing experimental data; and to propose a practical method to tune muscle control parameters. To the authors’ knowledge, the controllers based on the equilibrium point control hypothesis (Bayer et al 2017) in combination with the muscle stretch reflex patterns (Feller et al 2016) are best suited for MLF control tasks. These controllers are already well established in the field of biomechanics and have the potential to improve the application of AHBMs significantly, as they are omnidirectional and biofidelic in different types of simulations. Furthermore, they are biologically and physiologically motivated as the primary feedback control variable is the muscle spindle signal. Hence, such controllers are especially well suited for posture maintenance during or before an impact and open up the potential to simulate different occupant movements during manoeuvres, for example, steering. A faster calculation time in LS-DYNA can be achieved by including the controller in the material model implemented in the Fortran programming language (Wochner et al 2019). This approach saves a significant part of the computational resources spent on interpreting keywords and allows for the control of each muscle element separately, having access to all internal material variables if needed. The verification of the controller implementation is performed on a base of experimental data for arm motion (Kistemaker et al 2006), head–neck region reflexive behaviour (Wochner et al 2022), as well as posture stabilization during braking manoeuvre (Huber et al 2015), in combination with two freely available FE HBMs VIVA OpenHBM (Östh et al 2017a) and THUMS v5 (Iwamoto and Nakahira 2015). This enabled us to perform a comparison of different control strategies at the arm level and testing of the reflex controller at the neck and whole body levels. The muscle control parameters were tuned and transferred between similar musculoskeletal models during the verification process, and the entire procedure is described in the corresponding chapters. Finally, the source code for the material model with the controller is available open-source as supplementary material to this paper (Nölle et al 2022), intending to facilitate the work of developers and researchers using our muscle model in their AHBMs in the future.

## Materials and methods

### Elements of forward dynamics simulations of human motion

**Fig. 3:**
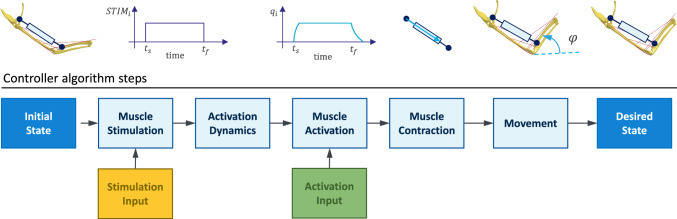
Generic flowchart of the movement simulation with finite element models with open-loop controller input. Note that only exclusive stimulation $STIM_{i}$ or activation $q_{i}$ input is possible, but not both at a time

An AHBM represents a muscle-driven forward-dynamics simulation model. The basic principle of its function is shown in Fig. 3. Several internal processes are involved to bring the model from the initial to the desired state. First, the controller sends stimulation or activation input signals depending on the desired position’s model parameters. In the case of an incoming stimulation input signal, it is transformed into muscle activation through activation dynamics. Second, muscle contraction occurs as a reaction to the activation signal, which results in a force generation between its insertion and origin points. Finally, movement is generated by the imposed joint torques, which occur from the sum of all muscle forces acting over a distance to the joint centre, and the model reaches its desired state. We will discuss all the processes, including the structure and function of the controller, in the following chapters.

### Functionality of the extended Hill-type muscle model

**Fig. 4:**
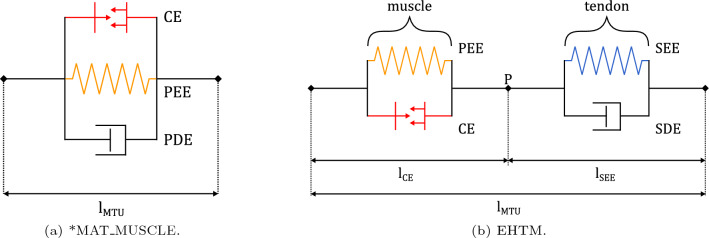
Schematic structure of the Hill-type muscle material models:** a** three element *MAT_MUSCLE available in LS-DYNA and** b** four-element extended Hill-type muscle model (EHTM), where CE—Contractile Element, PEE—Parallel Elastic Element, PDE—Parallel Damping Element, SEE—Serial Elastic Element, SDE—Serial Damping Element

The macroscopic Hill-type muscle model initially proposed by A.V. Hill in 1938 (Hill 1938) represents the whole three-dimensional muscle-tendon unit (MTU) with a simplified one-dimensional entity (element). This element comprises all properties of the MTU described by nonlinear dependencies of the output force over muscle length, contraction velocity and activation level. The respective Hill-type material model in LS-DYNA is called *MAT_MUSCLE and consists of three simple mechanical elements in parallel: contractile element (CE), parallel elastic element (PEE) and parallel damping element (PDE) as shown in Fig. 4a. Such a combination of internal elements has certain drawbacks according to the data published in (Kleinbach et al 2017). Namely, they are the tricky definition of material properties and parameters based on the anatomical literature and obscure approximation of the physical muscle structure. As a possible solution, we introduce an open-source extended Hill-type muscle (EHTM) material model consisting of four simple mechanical elements combined in two groups as seen from Fig. 4b: the contractile element (CE) together with the parallel elastic element (PEE) represents the active muscle fibres, whereas the serial elastic element (SEE) and the serial damping element (SDE) correspond to the characteristics of the tendon. In addition, the EHTM encapsulates muscle activation dynamics which transforms stimulation signal coming from the nervous system into muscle activation and is considered an inherent physical muscle function, see Fig. 3.

Since the current work focuses on implementing and verifying physiological muscle controllers into the EHTM, a short review of the basic model elements and their functionality is given in the Appendix A, with the detailed description available for the interested reader in previous publications (Haeufle et al 2014; Kleinbach et al 2017). Only the update made for the activation dynamics formulations in this contribution is described below.

#### Activation dynamics

The EHTM was extended by an updated version of the Hatze activation dynamics (Hatze 1977) proposed by (Rockenfeller and Günther 2018) during work on the current manuscript. In this formulation, the equation describing the $Ca{}^{2+}$ ions sensitivity of the muscle was simplified to:

1 $$\begin{aligned} \rho (l_{CE,rel}) = c \cdot \eta \cdot l_{CE,rel}, \end{aligned}$$

where $l_{CE,rel} = l_{CE} / l_{CE,opt}$ – relative length of the CE; *m*, *c* and $\eta$ – Hatze constants. It results in faster code execution with similar calculated values for the muscle activity.

It is recommended to use this version of activation dynamics for better performance and more adequate biofidelic response when running simulations with the EHTM based on the results reported in studies (Rockenfeller and Günther 2016, 2018).

#### Parametrization

**Fig. 5:**
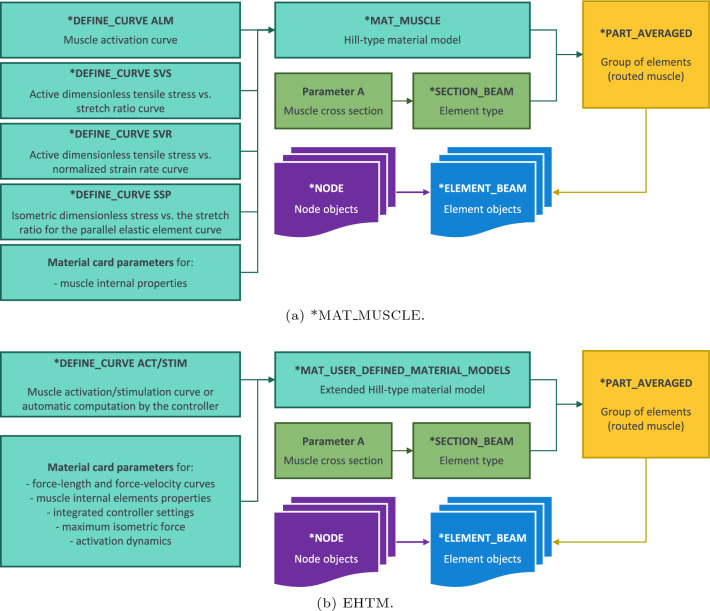
LS-DYNA objects hierarchy diagrams showing the difference between parametrisation of the *MAT_MUSCLE and the EHTM

Even though both the EHTM and *MAT_MUSCLE models are based on the same modelling approach representing the biological muscle with one-dimensional elements, they have different parameter definitions. As seen in Fig. 5a, parametrisation of the *MAT_MUSCLE is mainly based on pre-defined load curves and allows for the input of several internal muscle properties. The EHTM instead accepts curve inputs for muscle activation or stimulation signals and allows for the adjustment of the muscle properties by the input of scalar parameters addressing Hill-type material model properties, controller settings and activation dynamics as displayed in Fig. 5b. There is no need for the user to recompute and enter data points for load curves responsible for the force–length and force–velocity relations, as the EHTM calculates all this automatically during runtime based on the parameters input in Keywords. The EHTM parameters are divided into *non-specific*, which are more or less similar for various muscle groups, and *specific*, which are unique for each individual muscle. The non-specific muscle parameters are collected in Table 1. Muscle-specific parameters are model dependent and include the maximum isometric force $F_{max}$, the optimal fibre length $l_{CE,opt}$ and the tendon slack length $l_{SEE,0}$. Therefore, tables with muscle-specific parameter values are given in the appropriate sections of this publication. It is worth mentioning here that although the force at the nonlinear–linear transition $\Delta F_{SEE,0}$ is a muscle-specific parameter, it is not given in the appropriate tables as it can be derived from $F_{max}$ using the formula (Bayer et al 2017):

2 $$\begin{aligned} \Delta F_{SEE,0} = 0.4 \cdot F_{max}. \end{aligned}$$

**Table 1:** Non-specific muscle parameters from (Bayer et al 2017, Table 3 and Table 4) converted to the unit system of the VIVA OpenHBM model (kg-mm-ms-kN)

Act	Hatze parameters					Zajac parameters		
	$q_0$	c [$\frac{mol}{L}$]	$\eta$ [$\frac{L}{mol}$]	k	m [$\frac{1}{ms}$]	$q_0$	$\tau _{q}$ [*ms*]	$\beta _{q}$
	0.005	1.37e$-$4	52700.0	2.9	0.0113	0.005	25.0	0.5

CE	Force–length relation				Force–velocity relation			
	$\Delta W_{exp,des}$	$\Delta W_{exp,asc}$	$\nu _{CE,des}$	$\nu _{CE,asc}$	$A_{rel,0}$	$B_{rel,0} [\frac{1}{ms}]$	$F_{ecc}$	$S_{ecc}$
	0.45	0.45	1.5	3.0	0.2	0.002	1.5	2.0

PEE			SEE			SDE	
$\mathcal {L}_{PEE,0}$	$\nu _{PEE}$	$\mathcal {F}_{PEE}$	$\Delta U_{SEE,nll}$	$\Delta U_{SEE,l}$	$\Delta F_{SEE,0} [kN]$	$D_{SDE}$	$R_{SDE}$
0.95	2.5	2.0	0.0425	0.017	$0.4 \cdot F_{max}$	0.3	0.01

#### Routing capabilities

**Fig. 6:**
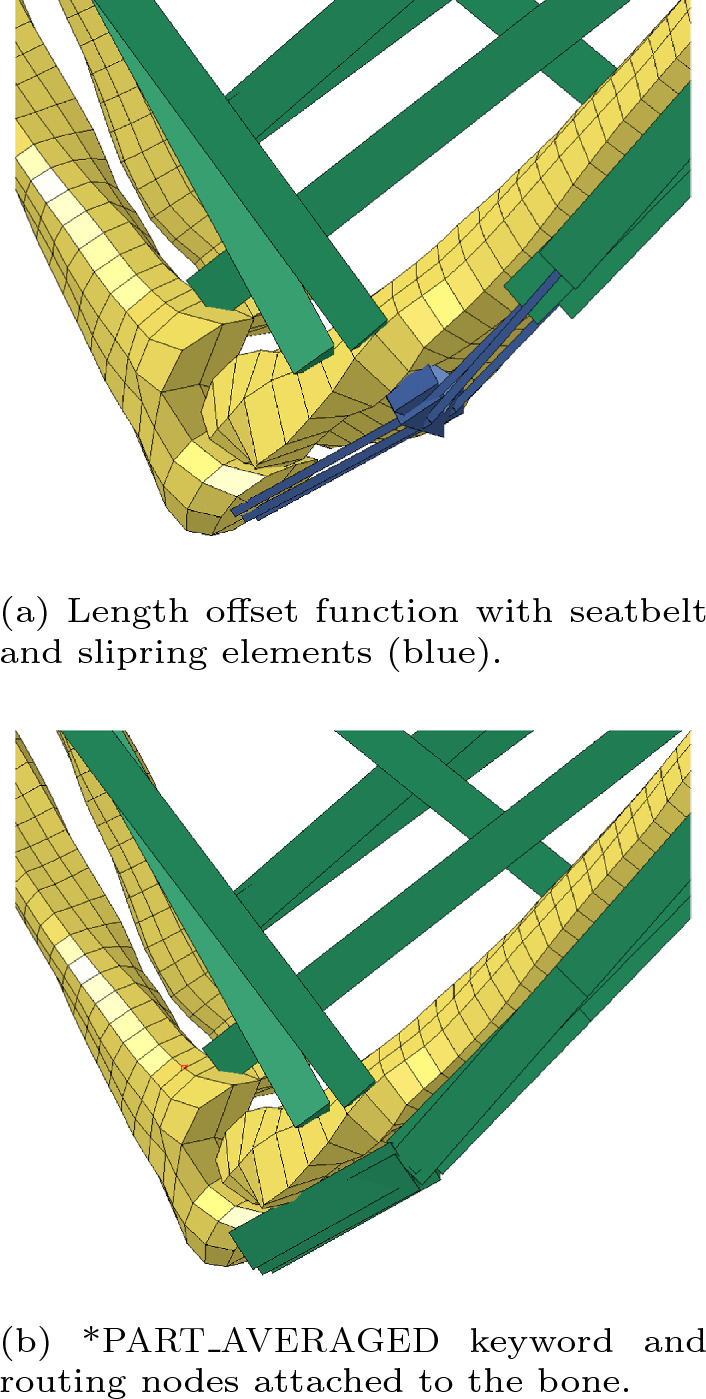
Close-up of the VIVA OpenHBM model elbow joint with the EHTM material truss elements (green) modelled using different routing approaches

Accurate modelling of muscle function in a human body is impossible without considering the correct line of action of each muscle. Muscle wrapping over joints determines the direction of force applied to the musculoskeletal system and affects the resulting muscle moment arms related to the centre of a joint. Therefore, the implementation of the physiological muscle path is crucial for obtaining valid results with AHBMs. Initially, we included the length offset routing functionality into the EHTM based on the fixed via-point method (Kleinbach et al 2017; Fehr et al 2017). To route the muscle when modelling utilizing the LS-DYNA functionality, it was necessary to build the construction of the EHTM and seatbelt *MAT_SEATBELT element attached to the model at a routing point with a slip ring *ELEMENT_SEATBELT_SLIPRING. During the further development process, it was discovered that such an approach introduces numerical instabilities into the AHBM due to several reasons connected with the FEA solution process (Martynenko et al 2018). Consequently, it is suggested to use the *PART_AVERAGED keyword for a single (non-branching) line of truss elements to wrap the muscle geometry (LSTC 2016). In such a case, all the connected EHTM elements are considered as one long continuous “macro-element” with an averaged strain and strain rate over the full length of the muscle. Correct muscle force transduction to the model can only be achieved, if the nodes used in defining the muscle path are properly constrained to the model at all insertion, origin and deflection points. Depending on the model, the authors propose to use the *CONSTRAINED_EXTRA_NODES, *CONSTRAINED_INTERPOLATION, or *CONSTRAINED_NODAL_RIGID_BODY keywords. A comparison of the different routing methods described above is shown in Fig. 6 on the example of the VIVA OpenHBM upper extremity. Comprehensive information about the other muscle routing methods for the Hill-type models and their influence on the musculoskeletal systems can be found in (Hwang et al 2016; Hammer et al 2019).

### Integrated muscle control strategies

**Fig. 7:**
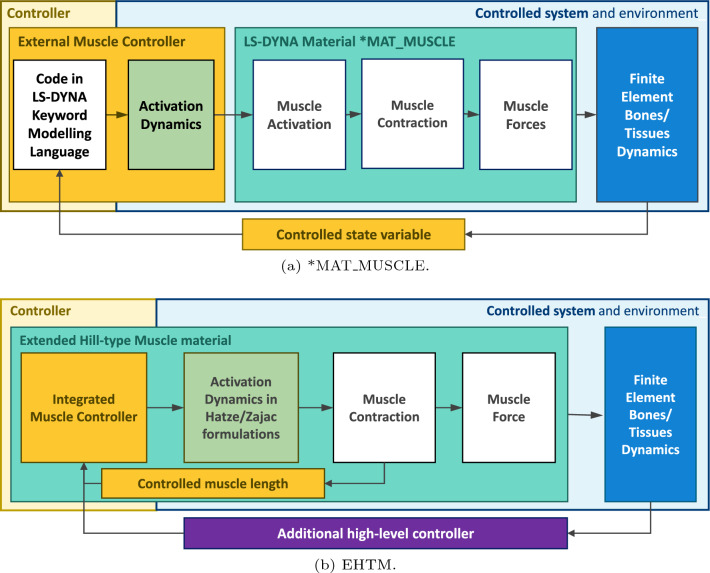
Interaction of the controller with the musculoskeletal model in LS-DYNA for different materials

Muscle control is a prerequisite for any simulation considering active or reactive AHBM behaviour. As mentioned above, the material model *MAT_MUSCLE existing in LS-DYNA does not provide any possibility to control muscle elements apart from code written in the keyword modelling language. Therefore, all modern AHBMs implemented in LS-DYNA contain an extensive amount of such controller code because of the need to control hundreds of muscle elements (Iwamoto and Nakahira 2015; Östh et al 2015; Devane et al 2019). Considering that keywords are interpreted during runtime, this leads to sluggish code execution and unnecessarily prolonged simulation times. In addition, some facts from muscle physiology are not taken into account in the *MAT_MUSCLE material formulation or not yet in the code of some controllers. For instance, a physical muscle can perform rudimentary feedback control tasks due to its physiological construction through its proprioceptors (Loeb and Mileusnic 2015). In addition, it simplifies the high-level neuronal control effort as compared to the torque actuators used, for example, in multi-body Madymo AHBM (Meijer et al 2013), due to its nonlinear characteristics (Haeufle et al 2020). Therefore, we suggest integrating the controller code written in Fortran directly into the open-source EHTM to overcome these issues (Fig. 7). As such, after a single compilation of the LS-DYNA binary, it will be executed with the maximum speed allowed by the operating system afterwards (Wochner et al 2019). Moreover, in contrast to existing technical muscle control approaches implemented in the AHBMs referenced above, we propose to incorporate the physiologically inspired equilibrium point control hypothesis (Feldman and Levin 2009) and the muscle length-based stretch reflex (Feller et al 2016; Latash 2021) as control strategies. Both control strategies replicate the usage of muscle proprioceptor sensory signals for a feedback control loop and are described in details below.

#### Muscle proprioceptors and their signals

**Fig. 8:**
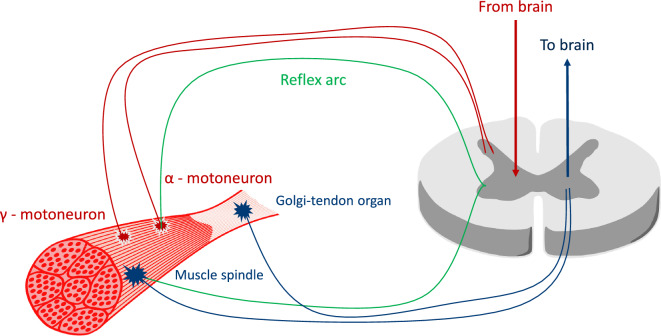
Neuromuscular junction—background for the physiological muscle controller

The MTU includes two types of specialized mechanoreceptors connected to the nervous system via afferent nerve fibres. They are the muscle spindles located in the muscle belly combined with extrafusal muscle fibres and the Golgi tendon organs, which can be found between the tendon and muscle fibres, see Fig. 8. The muscle spindle perceives variations in the muscle fibres length $l_{CE}$ and velocity $\dot{l}_{CE}$ due to the contraction or extension processes (Loeb and Mileusnic 2015). The Golgi tendon organs are sensitive to the muscle’s tension, having an output signal proportional to the tendon force $F_{SEE}$. The EHTM code calculates these variables during runtime; therefore, they are easily accessible by the proposed closed-loop controller function. Moreover, other signals produced by the controllers at a higher level, as shown in Fig. 2, can be easily added to the internal control approach.

#### Equilibrium point control theory

To the present day, a lot of different control concepts and hypotheses in the field of motor control have been proposed. Based on the author’s premise to target the lowest level of control possible, it makes sense to implement one of the control concepts that explicitly use the musculoskeletal system properties and proprioceptive feedback loops. The best suiting control theory is the equilibrium point control hypothesis (Kistemaker et al 2006; Feldman and Levin 2009; Bayer et al 2017). It is based on the assumption that the central nervous system governs the motion through shifting between particular states of the musculoskeletal system—“equilibrium points” (EPs). In these EPs, the equilibrium of all acting external and internal forces is maintained, while small perturbations are compensated by elastic and damping properties of the muscles. Moreover, it is shown that due to the intrinsic dynamics of the musculoskeletal system, it is possible to generate a movement with a smooth trajectory using several discrete EPs according to the intermittent control concept (Gawthrop et al 2018). The EP controller can be formulated in three different notations: an open-loop $\alpha$–controller, a closed-loop $\lambda$–controller and their combination—a hybrid controller. These formulations are discussed in the following.

$$\alpha$$–***Controller***

The $\alpha$–controller is a simple open-loop controller, which uses no sensory input from the proprioceptors. The output muscle stimulation signal is either defined before the simulation or is determined by any kind of an overlying controller during the simulation runtime and is formulated in Eq. 3.

3 $$\begin{aligned} STIM_{\alpha }(t) = STIM_{open}(t) \vert _0^1 \end{aligned}$$

$$\lambda$$–***Controller***

 The $\lambda$–controller is a closed-loop controller, which uses a signal from the muscle spindles as feedback. In other words, it can be described as a muscle length feedback controller that monitors the target length $\lambda$ defined by the EP. The other important variable in its formulation given in Eq. 4 is the neural or electromechanical delay (EMD) $\delta$ of the muscle contraction. It describes the time lag between the muscle stimulation and a measurable change in its force output, representing transient electrochemical and mechanical processes happening in the muscle during contraction (Cavanagh and Komi 1979). It is worth mentioning that the $\lambda$–controller represents a proportional-derivative (PD) controller. Equilibrium point control theory assumes that the function of an integral part in the PID controller is fulfilled by the intrinsic dynamics of the muscle model itself (Kistemaker et al 2006). Moreover, the velocity-dependent term with the derivative gain $k_d$ permits the choice of higher values for the proportional gain $k_p$ for the length-dependent term and stabilizes the output signal.

4 $$\begin{aligned}&STIM_{\lambda }(t, l_{CE}, \dot{l}_{CE}, \lambda , \dot{\lambda }) \nonumber \\&\quad = \left. STIM_{closed}(t, l_{CE}, \dot{l}_{CE}, \lambda , \dot{\lambda }) \right| ^{1}_{0} \nonumber \\&\quad = \left. \left\{ \frac{k_p \left[ l_{CE}\left( t - \delta \right) - \lambda \right] + k_d\left[ \dot{l}_{CE}\left( t-\delta \right) - \dot{\lambda }\right] }{l_{CE,opt}} \right\} \right| ^{1}_{0}, \end{aligned}$$

where $k_p$ is the proportional and $k_d$ is a derivative gains; $\lambda$ is the target length of the contractile element defined by the EP; $\dot{\lambda }$ is the target velocity of the contractile element contraction; $\delta$ is the neural or electromechanical delay (EMD) of the muscle contraction; $l_{CE,opt}$ is the optimal and $l_{CE}$ is the momentary muscle fibre lengths; $\dot{l}_{CE}$ is the momentary contraction velocity of the muscle.

***Hybrid controller***

 The hybrid controller is a combination of the open-loop $\alpha$– and closed-loop $\lambda$–controller. Such a combination suggested in (Kistemaker et al 2006) not only increases the velocity of the motion but also enhances the performance of the biomechanical system due to the addition of the velocity term, which provides nonlinear damping. The original formulation addressed in the following as “Kistemaker’s version” and shown in Eq. 5 assumes that the second term from the $\lambda$–controller can be both negative and positive. It accounts for the hypothesis that the musculoskeletal system movement is controlled from the superficial levels, see Fig. 2. Hence, such aspects as an inhibitory effect in an agonistic-antagonistic setup can be captured (Walter et al 2021):

5 $$\begin{aligned}&STIM_{hyb}(t, l_{CE}, \dot{l}_{CE}, \lambda , \dot{\lambda }) \nonumber \\&\quad = \left. \left\{ STIM_{\alpha }(t) {+} STIM_{\lambda }(t, l_{CE}, \dot{l}_{CE}, \lambda , \dot{\lambda }) \right\} \right| ^{1}_{0} \end{aligned}$$

The EHTM is designed to represent a distinct physical muscle reduced to a single finite element, containing a whole range of properties and functions, including simple internal control. It is known that the natural muscle can generate active force only when contracting. Therefore, to have a proper internal controller function, the target length $\lambda$ should always be less than the instantaneous muscle fibre length $l_{CE}$. Consequently, the “standard version” of the hybrid controller has the $\lambda$–controller addendum set to only positive as shown in Eq. 6. This formulation accounts for situations where no upper control levels are involved, and only the signals from internal proprioceptors govern muscle contraction:

6 $$\begin{aligned}&STIM_{hyb}(t, l_{CE}, \dot{l}_{CE}, \lambda , \dot{\lambda })\nonumber \\&\quad = \left. STIM_{\alpha }(t) \right| ^{1}_{0} + \left. STIM_{\lambda }(t, l_{CE}, \dot{l}_{CE}, \lambda , \dot{\lambda }) \right| ^{1}_{0}. \end{aligned}$$

***Reflex controller***

 Due to the integration of the controller inside the EHTM, it is possible to implement additional control strategies, which rely on internally available variables such as the lengths of muscle fibres $l_{CE}$ and the tendon slack length $l_{SEE}$. One such strategy is the muscle stretch reflex, also referred to as the monosynaptic stretch reflex, which is by nature a reaction of a muscle to external stretch in a longitudinal direction (Feller et al 2016; Latash 2021). Initially, the muscle-tendon complex acts completely passive and its elongation is mitigated by damping and elastic material properties of peripheral tissue. Only after a certain magnitude of length (strain threshold $\omega$), the muscle starts to react to further stretching by an increase in the activation level. The muscle spindles primarily govern such an activation. Hence, the stretch reflex functionality was added to the EHTM, as the authors believe that such a control strategy is helpful for posture maintenance in AHBM simulations. The reflex controller sets the stimulation signal $STIM_{reflex}$ to 0 or 1 based on the current muscle element strain. It is inactive until the simulation time *t* surpasses the sum of the controller activation time $t_{contr}$ set in a material control card plus time of the neural delay for muscle activation $\tau$ provided that the given strain threshold $\omega$ is exceeded. When activated, the contractile element strain $\varepsilon _{CE}$ is computed as follows:

7 $$\begin{aligned} \begin{aligned}&\varepsilon _{CE} (l_{CE,delay}) {=} \frac{ l_{CE,delay} {-} l_{CE,ref} }{l_{CE,ref}}, \\&where \quad l_{CE,delay} (t) {=} l_{CE}\left( t{-} \tau \right) . \end{aligned} \end{aligned}$$

In the following step, $\varepsilon _{CE}$ is compared with the strain threshold $\omega$ defined in the material card, and the constant stimulation signal *STIM* is consequently generated:

8 $$\begin{aligned} STIM_{reflex}(t, \varepsilon _{CE})= & {} 0, \; when \; \varepsilon _{CE} \le \omega , \end{aligned}$$

9 $$\begin{aligned} STIM_{reflex}(t, \varepsilon _{CE})= & {} 1, \; when \; \varepsilon _{CE} > \omega . \end{aligned}$$

Although the muscle stimulation signal generated by the reflex controller has a binary value, it results in a smooth and continuous activation signal due to the low-pass filter properties of the activation dynamics. We elaborate more on this topic in Sect. 4.3 Aspects of the Reflex Controller Implementation.

### Finding control parameters

In order to model voluntary motion and obtain accurate simulation results, one must use a precise muscle activation strategy. Although the equilibrium point control hypothesis does not require a-priory knowledge of the dynamics of the model, the underlying assumption of pre-learned movements is substituted by a data-driven optimisation. In particular, it is needed because normally the number of muscles in the musculoskeletal system exceeds the number of physical degrees of freedom, resulting in multiple combinations of the muscle activation levels corresponding to the same targeted equilibrium position (EP). Besides, all muscle controllers integrated into the EHTM have different parameters, some of which must be determined before the desired simulation runs by fitting of calibration experiments. In general, any optimisation method or tool available could be used to perform this procedure. In the current contribution, we used the software LS-OPT to find controller parameters and muscle activation vectors in the verification example because it is explicitly designed for parameter optimisation in LS-DYNA models.

#### Optimisation criteria and cost functions

The vector of muscle stimulation values $STIM_{open}$ which maps the joint angles to the desired EPs was obtained in LS-OPT by specifying additional constraints, optimisation criteria and cost functions. As a rule, EPs could be characterized by only one or several positional parameters. In our example, simulations of arm motion it is a constant elbow angle $\phi _{elb}^\text {target}$. The optimisation problem constraints for such a case ensuring a stable end position at $t=t_1$ can be formulated as follows

10 $$\begin{aligned} \vert \phi _{elb}(t_1)-\phi _{elb}^{target} \vert< & {} \epsilon _{\phi }, \end{aligned}$$

11 $$\begin{aligned} \vert \dot{\phi }_{elb}(t_1) \vert< & {} \epsilon _{\dot{\phi }}. \end{aligned}$$

Due to the redundancy of the musculoskeletal system, there are usually multiple sets of stimulation values that map to the target position. Therefore, additional optimisation criteria need to be formulated. Possible criteria are (1) the minimization of the sum of individual muscle stimulations, optimizing for control effort efficiency-based motion generation

12 $$\begin{aligned} \min \left(\sum _{i=1}^{N} STIM_{i}\right); \end{aligned}$$

or (2) the maximization of low-frequency joint stiffness, favouring positions with lower sensitivity against external disturbances (Bayer et al 2017)

13 $$\begin{aligned} \min (\phi _{elb}-\phi _{elb}^{disturb}). \end{aligned}$$

We suggest using the latter method and propose to apply a constant external force as disturbance after $t=t_1$ to evaluate the low-frequency joint stiffness by measuring the resultant change of position $\phi _{elb}^{disturb}$.

#### Motion generation

According to the equilibrium point control hypothesis, voluntary motion can be performed by switching between the EPs. Each EP can be characterized by the optimised vectors $STIM^{opt}$ for the $\alpha$–controller and by the corresponding muscle fibre lengths $l_{CE}=\lim _{t \rightarrow \infty } l_{CE}(STIM^{opt})$ for the $\lambda$–controller (so called target lengths $[\lambda ]$). Therefore, the following mapping scheme

14 $$\begin{aligned}{}[STIM^{opt},l_{CE}] \mapsto \phi _{elb} \end{aligned}$$

allows for the application of $\alpha$–, $\lambda$– and hybrid controller formulations to perform the transition from one EP to another. Motion is generated by changing optimised stimulation values, corresponding muscle fibre lengths $[l_{CE}]$ or both value sets combined in the controllers input at certain time steps during runtime.

### Verification

Verification of the EHTM updated code and implemented controller strategies was done using simulations with different models, which were created based on the THUMS v3 50th percentile male (Iwamoto et al 2007), the THUMS v5 50th percentile male (Kato et al 2017) and the VIVA OpenHBM 50th percentile female (Östh et al 2017a) full-body FE HBMs. Simulations were conducted based on a simulation matrix given in Table 2 built with an intention to verify both the passive behaviour and all the controller strategies available. First, the passive elastic and damping properties for the EHTM and *MAT_MUSCLE were compared based on THUMS v5 and verified with the experimental data for 50 km/h emergency braking manoeuvre from the Occupant Model for Integrated Safety (OM4IS) project (Huber et al 2015). Second, integrated control strategies were tested on the upper extremity models extracted from the VIVA OpenHBM and the THUMS v3 (Iwamoto et al 2007) HBMs and verified with the experimental data from (Kistemaker et al 2006). Finally, the reflex controller was verified with the “Falling Heads” experimental data from (Wochner et al 2022) based on the VIVA OpenHBM. Information on the original models, their appearance and main characteristics are provided in the Appendix D Table 3, followed by a detailed description of model and simulation set-ups. As the THUMS v3 is a passive HBM without muscle elements and a precursor of the THUMS v5, which is an improved updated version of it, and only an arm from it was used for simulations, the latter one was not included into Table 3. All the simulations were run in LS-DYNA SMP and MPP version R9.3.1 (LSTC, Livermore, USA) compiled with the additional user subroutine for the EHTM. Results were pre- and post-processed using different versions of LS-PrePost (LSTC, Livermore, USA), HyperView (Altair, Michigan, USA), MATLAB (MathWorks, Massachusetts, USA) and Microsoft Excel (Microsoft, Redmond, USA). The simulation results for emergency braking and falling heads experiments were evaluated using the CORrelation and Analysis (CORAplus) software version 4.0.4 (Thunert 2017). Global evaluation settings for the interval of evaluation, corridor and cross-correlation, as well as the weighting factors, were kept default as suggested in the CORAplus manual. Results obtained with the upper extremity models were assessed using the mean squared error (MSE) method.

**Table 2:** Simulation matrix of the study

Load case	Emergency braking	Fast elbow joint movements	Stretch reflex of the cervical muscles
Published in	(Huber et al 2015)	(Kistemaker et al 2006)	(Wochner et al 2022)
Verified Functionality of the EHTM	Passive elastic and damping properties	Integrated control strategies	The reflex controller
FE model used	THUMS v5 full body	THUMS v3 and VIVA OpenHBM extracted arms	VIVA OpenHBM full body
Muscle material used	EHTM and *MAT_MUSCLE	EHTM	EHTM
Verified with	Experiment, EHTM vs *MAT_156	Experiment	Experiment
Compared variables	Displacements, head COG acceleration, neck angle	Elbow angle, angular velocity	Displacements

## Results

### Emergency braking

**Fig. 9:**
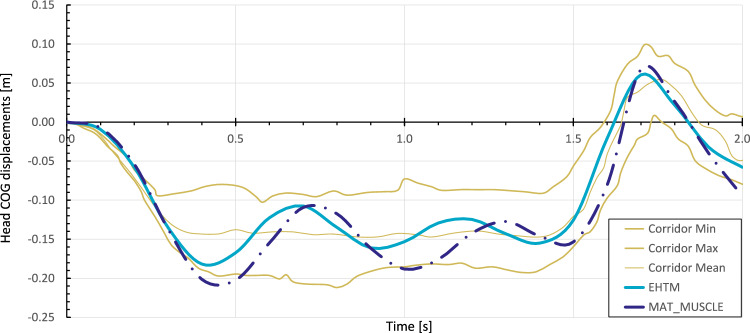
Displacement curves of the head’s centre of gravity (COG) in the sagittal plane during the 50 km/h OM4IS braking load case for *MAT_MUSCLE and the EHTM with positive displacements in posterior and negative displacements in anterior direction

**Fig. 10:**
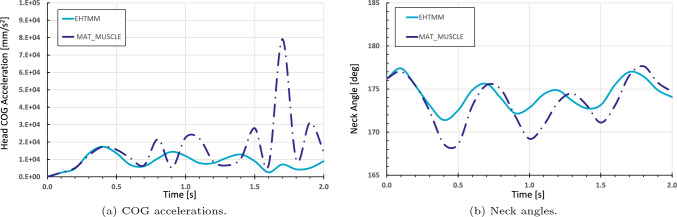
Comparison of the head–neck-kinematics during the 50 km/h OM4IS braking load case for for *MAT_MUSCLE and the EHTM.** a** Acceleration curves of the head’s centre of gravity (COG).** b** Neck angles, measured as the angles between the centres of gravity of the C7 vertebra, the C3 vertebra and the head’s COG

The emergency braking simulation results, see Fig. 9, show that the head COG displacement of the THUMS v5-EHTM model matches the displacement corridor of the volunteer experimental results well throughout the entire braking event. In contrast, the original THUMS v5 model produces head displacements which dip below the lower volunteer corridor, indicating that the inclusion of the EHTM material improves the kinematic response of the original model. Corresponding CORAplus ratings are 0.736 for the original THUMS v5 and 0.836 for THUMS v5-EHTM. Consequently, both models ensure good biofidelity with the THUMS v5-EHTM outperforming the default model by a score greater than 0.1 without any additional modifications. A more detailed comparison of the two models’ head–neck-kinematics shows that both the head accelerations Fig. 10a and the change in neck angle Fig. 10b are less pronounced for the THUMS v5-EHTM, showing that the passive properties of the EHTM alone are able to better stabilize the head during highly dynamic breaking scenarios. This can be explained by the different structures of two Hill-type models utilized (see Fig. 4b), which leads to slightly different mechanical properties. *MAT_MUSCLE is generally less compliant, as all of its three elements are connected in parallel, so that the PEE and the PDE are simultaneously producing forces during both extension and flexion of the muscle. The EHTM, on the other hand, is less stiff for low muscle strain rates for two reasons. First, the PEE force depends on the force of the CE, resulting in a lessened additional force output during extension under the external load (see Fig. 16b). Second, the SDE produces forces depending on the muscle strain only if the tendon has been stretched beyond its resting length (see Eq. A9) unlike the PDE of *MAT_MUSCLE which acts during all muscle elongations. Conversely, for high muscle strain rates, the EHTM will show a higher stiffness compared to *MAT_MUSCLE as all three of its elements (PEE, SEE, SDE, see Fig. 4a) will counteract the elongation of the MTU. The intrinsic low acceleration compliance of the EHTM could therefore be used to solve the issue of disproportionate HBM stiffness described in (Shelat et al 2016).

### Fast elbow joint movements

**Fig. 11:**
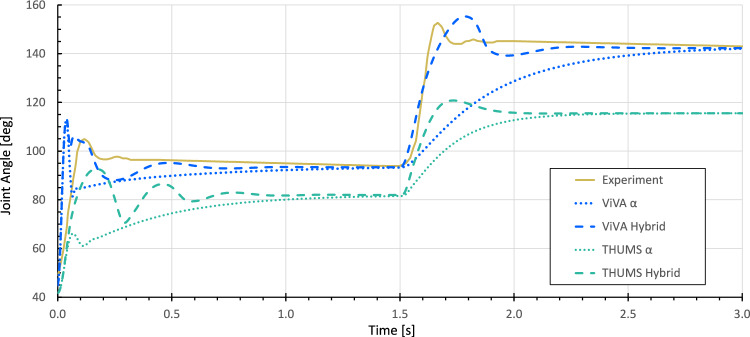
Joint angles of the VIVA OpenHBM and THUMS v3 arm models for $\alpha$– and hybrid controllers. Experimental results from (Kistemaker et al 2006) are given for the reference

**Fig. 12:**
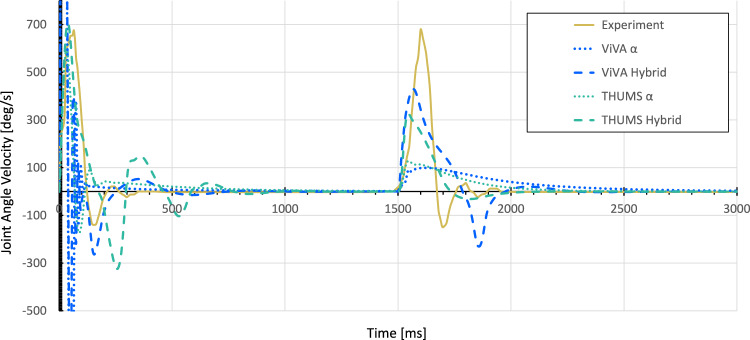
Angular velocity of the VIVA OpenHBM and THUMS v3 arm models for $\alpha$– and hybrid controllers. Experimental results from (Kistemaker et al 2006) are given for the reference

The resulting kinematics and associated joint angular velocity of the fast elbow joint movement simulations, displayed in Figs. 11 and 12, confirm the original hypothesis from (Kistemaker et al 2006), where it is stated that a combination of open-loop $\alpha$– and closed-loop $\lambda$–controller strategies leads to faster arm movement and a shorter control period than isolated $\alpha$– or $\lambda$–controller approaches. The second step response from 1.5 s is particularly suitable for direct comparison, as in all cases a preloaded system can be assumed here. The data displayed show both the shorter settling time in the simulations and an effective increase in the maximum velocity by more than a factor of 2.5 for both arm models. At the same time, the resulting movements obtained with the EHTM are still slower compared to the experimental results. This can explained by the fact that the current contribution’s main aim was to verify the implemented code but not to find perfect parameters sets for the controller. Therefore, the given results represent just one possible parameter set of control signals in a redundant system that has not been optimized further beyond the open-loop stimulation level. The simulation results for this set-up are evaluated by the mean squared error (MSE) method. The MSE for the ViVA OpenHBM arm simulations with optimised open- and closed-loop controller parameters (hybrid controller) yields a value of 19.04, when for open-loop it is only 276.45. For the THUMS v3, errors are more significant as controller parameters were not optimised but calculated with the scaling method described in Sect. 4.4 Parameters Transfer Between Different Musculoskeletal Models equalling to 818.59 for the single acting $\alpha$–controller and 549.47 for the hybrid one. Furthermore, the remaining offset in the end position between the kinematic results of the two arm models used for the simulations at 1500 ms and 3000 ms can be attributed to the difference in their individual body segment proportions. According to the workflow proposed above, the muscle length-based $\lambda$–controller signals defined for the VIVA OpenHBM-based model were scaled to the different muscle lengths of the THUMS v3 model, not considering the force-dependent lengthening of the serial elastic tendon element $l_{SEE}$. The results further demonstrate that the time-efficient transfer of muscle target lengths and the redefinition of stimulation values from hybrid control results from the previous step did not introduce instability or other model misbehaviour even for much stronger muscles with higher maximal isometric force as present in the male THUMS v3 arm model. Of course, the joint angles will generally not be met precisely with the suggested approach, but it can be a good starting point for further optimisation.

**Fig. 13:**
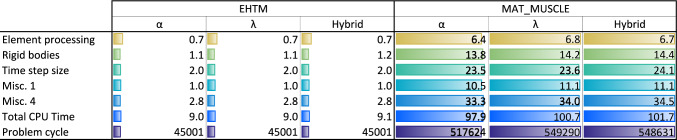
Comparison of the CPU time needed for the simulations with the EHTM and *MAT_MUSCLE for different controller strategies. Figure adapted from (Wochner et al 2019)

Furthermore, the upper extremity model based on the THUMS v3 and the same load case was used to evaluate the anticipated increase in runtime performance for the EHTM. Such expectations come from the fact that the controller is implemented as a FORTRAN subroutine and fitted into LS-DYNA software code. Hence, it should run much faster comparing to the controller code programmed with standard Keywords. Three different control strategies available in the EHTM were compared: (1) an open-loop $\alpha$–controller; (2) a closed-loop $\lambda$–controller; and (3) a combination of both—a hybrid controller. Controller neural delay was not accounted for Simulations that were performed on a workstation with the AMD Ryzen Threadripper 1950X processor and similar LS-DYNA starting options. A comparison of the CPU times with the exact computational load distribution over solution steps is shown in Fig. 13. As seen, due to the significant increase in muscle controller keywords processing for*MAT_MUSCLE (Misc. 4 row), all other simulation steps also require up to ten times more computational time even for such simple arm models. It should be noted here that controller’s time delay was not activated for these simulations. It is implemented through a memory ring buffer, thus consuming additional computational resources. Therefore, the authors expect that for real-world simulations with more sophisticated models and all EHTM options activated speed up of simulations would not be so significant.

### Stretch reflex of the cervical muscles

**Fig. 14:**
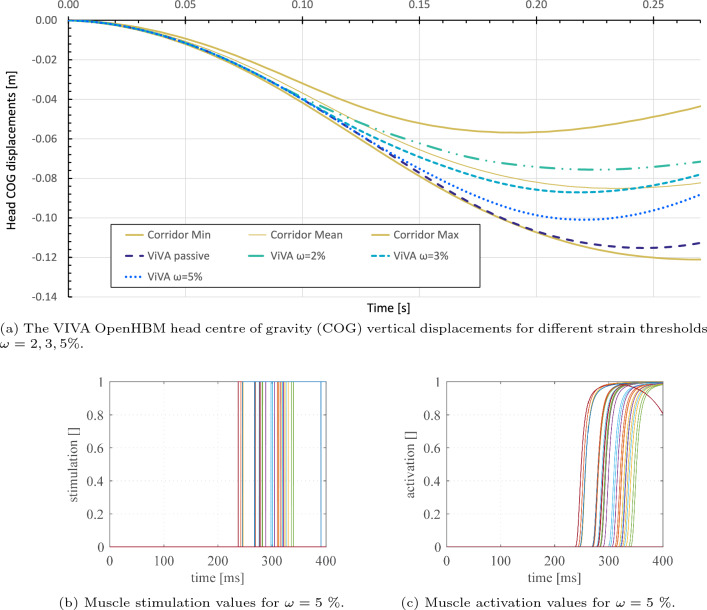
Simulation results for the stretch reflex of the cervical muscles of the VIVA OpenHBM model. The simulation time until complete model relaxation is excluded from graph (**a**)

The VIVA OpenHBM was used to verify the reflex functionality of the EHTM. The material of all its muscle elements in the head–neck region was swapped to the EHTM. The reflex controller was activated to stabilize the head and to decrease its total vertical displacement replicating the design of the “Falling Heads” experiments (Wochner et al 2022) as described in Appendix D.3. Simulation results are shown in Fig. 14a. As seen, the lower levels of strain threshold lead to earlier muscle activation and thus to decreased head displacements. The reflex controller ensures a gradual activation of the musculature from zero to maximum when surpassing the strain threshold limit $\omega$ to stabilize the head. Example values obtained in simulations with the threshold $\omega ={5}\%$ for each muscle are given for the stimulation in Fig. 14b and for the activation in Fig. 14c. The smooth activation signal illustrates the proper function of the activation dynamics integrated into the EHTM.

The CORAplus ratings of the “Falling Heads” simulations are 0.702 for the passive VIVA OpenHBM model, 0.904 for the strain threshold $\omega ={2}\%$, 0.987 for threshold $\omega ={3}\%$ and 0.854 for threshold $\omega ={5}\%$. Hence, the results obtained with the passive model and with threshold $\omega ={5}\%$ ensure good model biofidelity, whereas strain thresholds $\omega ={2}\%$ and $\omega ={3}\%$ correspond to the excellent biofidelity, with $\omega ={3}\%$ being the best. These findings are in accordance with the study (Putra et al 2020), which states that utilizing a simple PD controller at a muscle level to mimic cervicocollic reflexes shows good performance in stabilizing the head–neck region during loads that typically occur during rear-end impacts. Hence, a stretch reflex controller of the EHTM can be used for head–neck stabilization in crash scenarios when supplemented by an additional control function modelling the vestibulocollic reflex as suggested in (Correia et al 2021). Due to the short latencies, the stretch reflex allows for the generation of muscle force that influences head kinematics significantly, even during highly dynamic events. Therefore, this cervicocollic reflex is a credible source for the rapid response in muscle activity observed in experimental data and can be easily applied in simulations by defining the strain limit $\omega$ in the EHTM.

## Discussion

### Limitations

#### Material parameters uncertainties

As is the case with all material models, the correct choice of muscle material parameters is paramount for achieving physiologically valid results using the EHTM. In this study, the authors attempted to solve this issue on a case-by-case basis for each of the presented verification simulations. Wherever possible, muscle routing paths and force characteristics were reused from the original models. These parameters were originally determined and validated by the respective creators of the base models (Kato et al 2017; Östh et al 2017a). The other muscle-specific parameters ($l_{CE,opt}$ and $l_{SEE,0}$) were derived from anatomical literature. The generic muscle parameters were chosen according to the sensitivity analysis results provided by (Bayer et al 2017). The EHTM material parameters are thus in good agreement with both the parameters originally chosen by the creators of baseline models, as well as the muscle parameters of other EHTM implementations such as the models created for the well-established biophysics simulator demoa (Schmitt 2022). While the authors are thus confident that the chosen EHTM parameters are valid, no efforts have been undertaken to see if a retuning of the pre-set *MAT_MUSCLE material properties could have improved the observed model behaviour. In addition, the parameter tuning method used in the presented work (Wochner et al 2022; Nölle et al 2022) is one of the possible methods to achieve a sensible parameter set, from many approaches available (Heinen et al 2016; Rockenfeller et al 2020) Despite this, the inherent mechanical properties of the four element the EHTM make it a more numerically stable (Yeo et al 2023) and better represent the eccentric force-velocity relation of the biological muscle, when compared to three element Hill-type materials such as *MAT_MUSCLE (Günther et al 2007; Haeufle et al 2014).

An added benefit of the EHTM is the representation of a distinct muscle and tendon parts for each muscle element. This allows for the in-depth study of forces and strains acting on both parts of the MTU, for example for the assessment of muscle injury severity as was performed by (Nölle et al 2022). Moreover, the EHTM has been extensively studied in the context of multi-body simulations by other authors, who have performed exact comparisons of material behaviour with experimental data (Günther et al 2007; Mörl et al 2012). Similarly, in (Kleinbach et al 2017) it was shown that the LS-DYNA implementation of the EHTM is able to reproduce concentric, isometric, and quick-release experimental data derived from pig, rat, and cat muscles. The sensitivity of results achieved with Hill-type muscles has been studied extensively as well (Scovil and Ronsky 2006; Bayer et al 2017; Yeo et al 2023). Based on these findings, the authors are convinced that the EHTM can deliver physiologically valid results as long as sensible material parameters have been provided.

#### Combination with 3D muscle materials

Hill-type muscles are a phenomenological description of the skeletal muscle, which are useful in describing and studying the function of and control concepts applied to human and animal musculature (Caillet et al 2022). Despite these advantages, some aspects such as volumetric shape or the shift of muscle mass during contraction cannot be properly modelled using Hill-type models such as the EHTM. Thus, recent studies have focused on the creation of constitutive three-dimensional muscle models to describe the processes and effects of muscle contraction in more detail (Almonacid et al 2022; Saini et al 2022; Zeng et al 2023). While these models thus offer a more wholistic perspective on the topic of skeletal muscle behaviour and have shown to achieve more realistic results than their discrete, on-dimensional counterparts in some cases (Hedenstierna and Halldin 2008), one critical downside concerning their widespread use remains unresolved. The added complexity in the modelling of the musculature comes at a large increase in computational effort, resulting in unfeasibly long computation times when applied to full-body systems such as the AHBMs for which the presented implementation of the EHTM is designed as discussed in (Caillet et al 2022; Almonacid et al 2022). Future studies with the EHTM might focus on the combination of one-dimensional Hill-type elements with three-dimensional muscle structures to evaluate the possibility of combining numerical efficiency with a more realistic volumetric behaviour of the muscle following the logic of (Hedenstierna and Halldin 2008; Röhrle et al 2016).

### Improved numerical stability of the EHTM

**Fig. 15:**
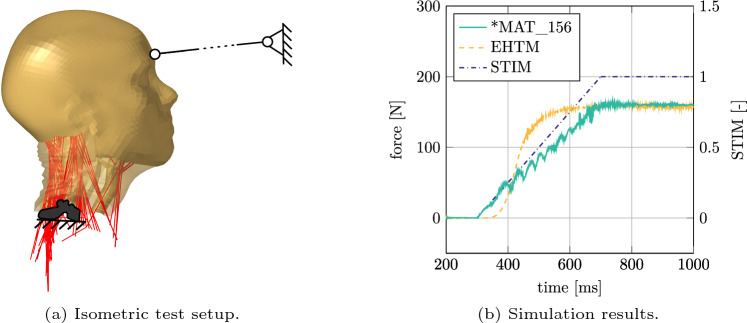
Isometric test setup with VIVA OpenHBM head–neck model after reaching the maximum force at $t=1000$ ms (**a**) and time dependant values generated during simulation (**b**). The stimulation values used to activate neck extensors (dash-dotted blue) and the resulting forces which occurs at the boundary for the EHTM (dashed yellow) and *MAT_MUSCLE(*MAT_156) (solid green)

**Fig. 16:**
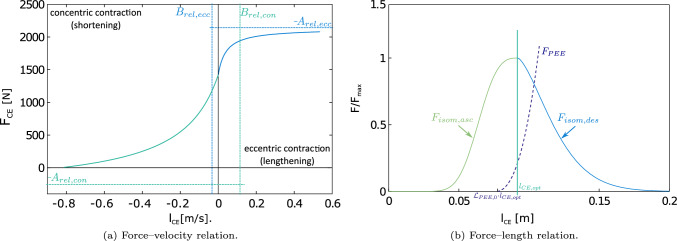
Exemplary force-velocity (**a**) and force-length (**b**) relations of an EHTM contractile element

An isometric load case with the isolated VIVA OpenHBM head–neck submodel (Östh et al 2017b) was used to rate the numerical stability of simulations with different Hill-type materials utilized in this study. In this setup, the model’s head was fixed using a beam element as shown in Fig. 15a. After a short initialisation phase $t \in [0,300]$ ms, the neck extensors were activated using a predefined stimulation signal rising from 0 to 1 within time interval $t=[300,700]$ ms (Fig. 15b). Integrated activation dynamics was enabled for the EHTM, while a pre-computed curve with the activation signals was used for *MAT_MUSCLE. As seen from the results in Fig. 15b, the EHTM and *MAT_MUSCLE show different characteristics in terms of force generation in the isometric test case. First, the forces measured in the beam element show a different onset behaviour. This can be explained by the more sophisticated four-elements composition of the EHTM compared to three-elements of *MAT_MUSCLE (see Fig. 4). Second, the force in the *MAT_MUSCLE simulations shows notable oscillations, which can be attributed to the absence of a serial damping element in the Hill-type material formulation that has proven to cause unphysiological oscillations (Günther et al 2007). Moreover, such oscillations can lead to instabilities in a FE solver and thus to increased computational costs in FE simulations. Hence, both aspects discussed indicate a higher biofidelity and an increase in numerical stability of the EHTM compared to *MAT_MUSCLE.

### Aspects of the reflex controller implementation

The reflex controller integrated into the EHTM in this study is based on the muscle stretch magnitude and generates binary control signals 0 or 1 corresponding to the muscle stimulation. Therefore, it could be considered as a bang-bang controller at this level. As shown in Fig. 3, the stimulation signal is transformed by the activation dynamics into a muscle activation signal leading to the muscle contraction afterwards. Hence, the activation dynamics act as a low-pass filter here, which means that even if a controller steps from zero to full muscle stimulation, the resulting activation signal will be smooth and continuous. It was proven during the EHTM verification process and can be seen for example in the transformation of the step-wise stimulation signal in Fig. 14b to the continuous activation signal in Fig. 14c. Moreover, in several other publications it was shown that some types of natural movement can be explained well by using such a piecewise-constant signal (Ben-Itzhak and Karniel 2008; Leib et al 2020). It is hypothesized that the underlying biological reason behind this is the reduction in the problem’s complexity, which in turn reduces the amount of information needed by the controller (Haeufle et al 2020). Based on this literature, we think that using such type of the reflex controller is appropriate in the automotive safety field where natural human movements are simulated.

Furthermore, we provide a verification example for the cervical muscle to support our statement. Simulation results show that having only the EHTM implemented in the model without any additional controller is enough to model the behaviour of the head–neck complex under gravity load correctly. In such a way, we can have an equivalent of the cervicocollic reflex without any need for additional controller code in LS-DYNA keywords, which is a substantial benefit compared to the earlier studies (Putra et al 2020; Correia et al 2021). In addition, the EHTM supports input of external control signals in parallel to the ones calculated internally. This functionality opens up the ability to model different types of reflexes at any part of the human body.

### Parameters transfer between different musculoskeletal models

Forward dynamic simulation of the muscle driven motion can be generally used to perform new movements or to mimic the existing others. In the current contribution, three different load cases based on existing experiments (OM4IS emergency braking (Huber et al 2015), “Falling Heads” experiment (Wochner et al 2022) and fast arm movements (Kistemaker et al 2006)) were shown that contributed to the topic of motion mimicking. The last load case has the broadest range of motion, where the final end-effector position needs to be deliberately moved away from the starting position. Such tasks are challenging, especially when there is no experimental data available to recalculate the particular motion inversely or when the model is functionally unknown. In that case, a machine learning (ML) method may be appropriate to define a possible set of muscle stimulation values, for example, as proposed in (Driess et al 2018). The general drawback of ML methods is high time consumption, which is highly dependent on the complexity of the muscle-driven system, the degree of redundancy, and the motion task posed. As a possible solution, the simulation task could be significantly simplified when similar musculoskeletal models are used for modelling. For example, if given models perform the same movement or task, but they have varying body sizes, muscle development levels, ages or a different gender with corresponding differences in anthropometric measures. We postulate that there is no need to revise the previously gained knowledge and learn an entirely new task in such a case. Instead, the previously acquired knowledge about the controller from one model can be transferred to the other to optimise the “learning process” with regards to time and resources.

The hybrid controller integrated into the EHTM allows two types of inputs: (1) an open-loop muscle stimulation signal that directly leads to an activation of the muscle and (2) a closed-loop signal based on the muscle length feedback. Direct re-application of the $\alpha$–controller signals alone in various models is impossible since differences in the achieved maximal active forces or the passive acting forces would automatically change the movement behaviour. In contrast, the $\lambda$–controller signals (contractile elements’ target lengths) can be easily transferred when scaling the values to the new initial muscle length of a similar model. In this way, comparable systems can mimic movements efficiently since potentially faster, overshooting, or slower behaviour would be compensated by the system morphology itself due to the difference in the maximum isometric muscle force, passive damping and elastic properties, and via a muscle length feedback loop. However, initial heuristic or ML-driven optimisation is still needed to define the initial set of target lengths for the muscles. This can be explained by the fact that there is no more straightforward option in the simulation environment to transform motion intentions from a higher level of control to a lower level as described in (Walter et al 2021).

As a limitation of the proposed method, we could mention that it is only suitable for mimicking movements with similar musculoskeletal models performing identical movements. Any changes in the muscular structure like muscles regrouping; adjusting deflection, insertion or origin points; or changing joint locations lead to a redetermination of the optimisation target. To prove our hypothesis, we performed the controller parameters transfer from the 50th percentile female VIVA OpenHBM to the 50th percentile male THUMS v3 model. The received results are shown in Fig. 11. As seen, there is a noticeable difference in results that increased with broader ranges of motion. Although the number of muscles and the associated representation in the models were identical, they had slight differences in the localisation of the applied action points, initial kinematics and body proportions. However, since the primary purpose was to demonstrate the advantages of the hybrid controller and its implementation in the EHTM, the results can be considered adequate. Taking converted $\lambda$–controller signals as the starting point for the following optimisation will significantly decrease the computational costs to find suitable input sets for reaching desired EPs.

## Conclusions

In this paper, we have implemented a physiologically motivated controller to the open-source extended Hill-type muscle (EHTM) material model for LS-DYNA and verified it with three different experimental datasets. The proposed controller operates with several control strategies based on open-loop and closed-loop methods or their combinations. Besides, it enables faster runtime and extendability through external high-level controllers. The suggested controller allows for the application of the EHTM in different load cases and under various conditions in automotive safety. However, to achieve the best results when using EP controllers, the user should still utilise an optimisation process to find activation levels for the open-loop controller part and do extensive work to find the proper contractile element’s target lengths. Furthermore, the application of the reflex controller requires adjustments of strain thresholds for each muscle individually to replicate the muscle onset behaviour observed in the experiments. Therefore, further research should be undertaken to investigate the appropriate muscle activation schemes and optimisation methods to find better strategies for all controller types applications. Moreover, it would be interesting to assess the effects of adding supplementary closed-loop feedback based on the Golgi tendon organ’s signal proportional to the force developing in the tendon tissues $F_{SEE}$ to the EHTM, or to supplement the stretch reflex controller with the vestibular one by adding the external stimulation $STIM_{open}$ input to model complex driving scenarios.

### Supplementary information

Below is the link to the electronic supplementary material.

Supplementary file 1 (pdf 108 KB)

## Figures and Tables

**Fig. 17:**
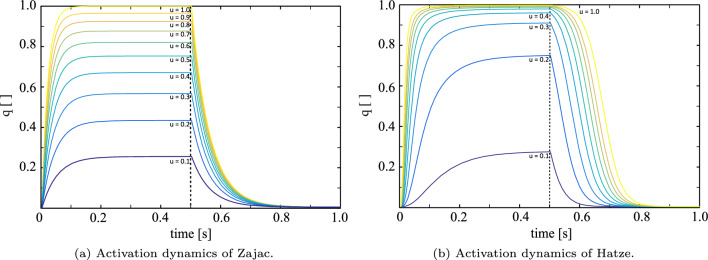
Comparison of the muscle activity level *q* over time for Zajac’s (a) and Hatze’s (b) activation dynamics for different stimulation levels *STIM* ranging from 0.1 to 1.0, wherein signal generation starts at 0.0 s and ends at 0.5 s. As seen, for high muscle stimulation, the time of increase in activity is shorter for Hatze’s activation dynamics than for Zajac’s, while the time of decrease in activity is longer. At low stimulation levels, the exact opposite applies

**Fig. 18:**
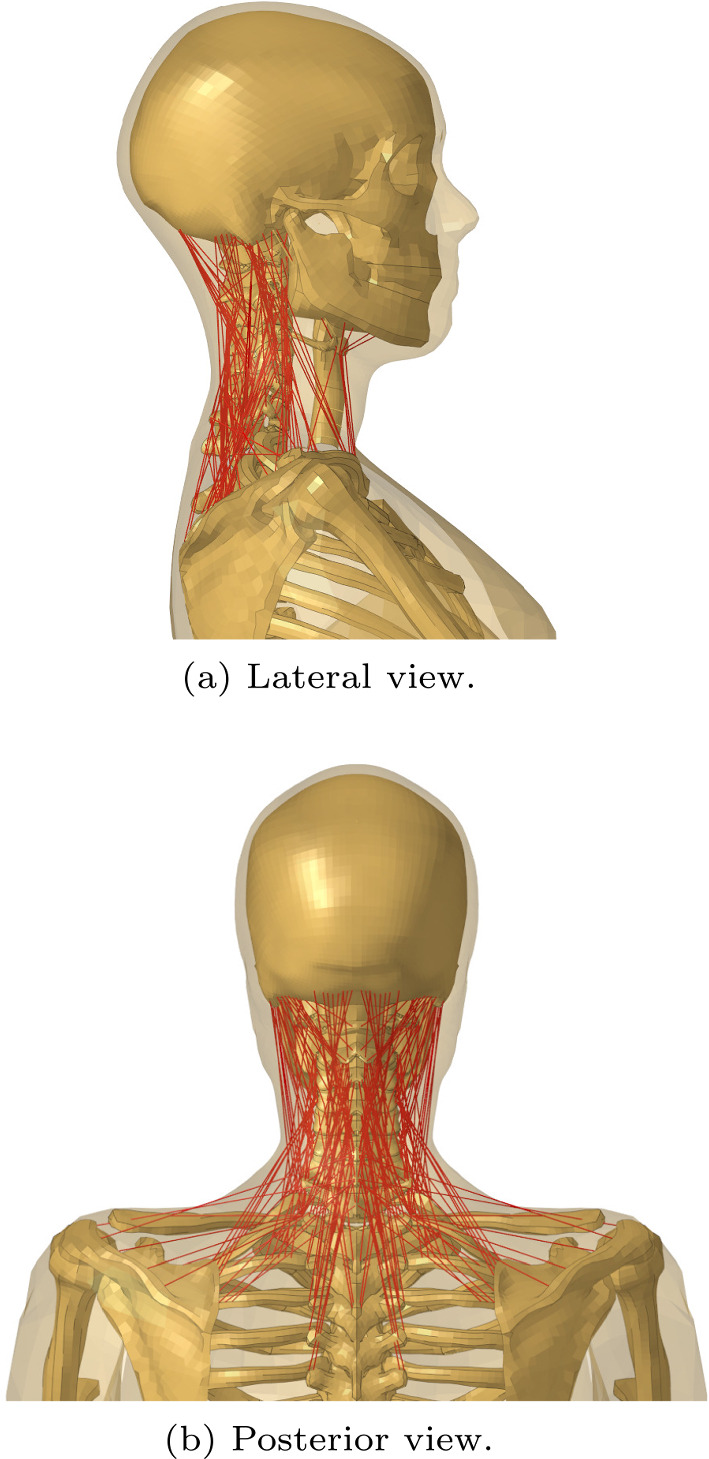
Neck muscles implemented in the VIVA OpenHBM

**Fig. 19:**
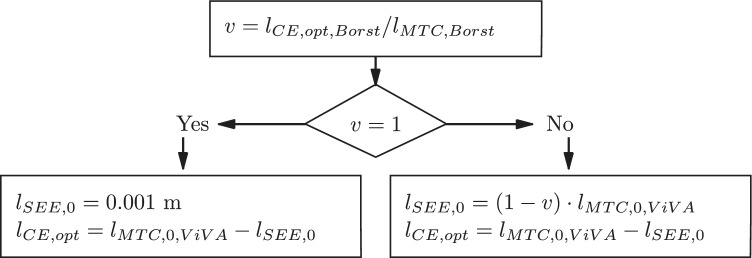
The algorithm for determining the optimal fibre length for the EHTM. It is based on the muscle element length in the VIVA OpenHBM model and accounts for the tendon length from the anatomical data (Borst et al 2011)

**Fig. 20:**
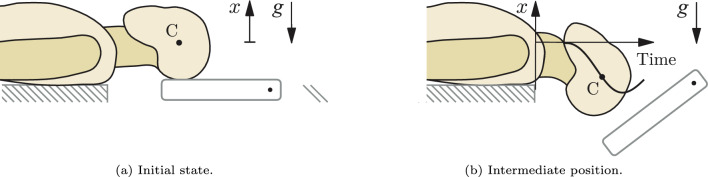
Schematic representation of the “Falling Heads” experiment from (Wochner et al 2022). Initial state (**a**) versus intermediate position with the head falling under gravity during experiment (**b**)

**Fig. 21:**
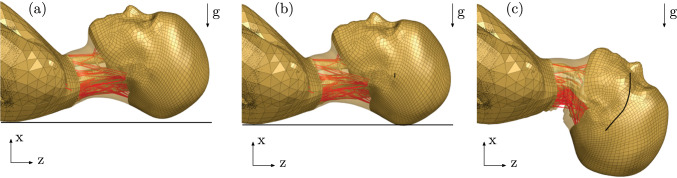
“Falling Heads” experimental set-up with the VIVA OpenHBM model in LS-DYNA.** a** Model position before the pre-simulation.** b** Model position after the pre-simulation which corresponds to the experiment start.** c** Model position at the lowest displacement point

## Data Availability

The following materials are available to supplement the manuscript: The EHTM Fortran code for the LS-DYNA umat41 subroutine at DaRUS (10.18419/darus-1144). The EHTM Material Cards Description and Corresponding Symbols at DaRUS (10.18419/darus-1144). Comments on the EHTM code implementation and compile instructions for the Fortran programming language at DaRUS (10.18419/darus-1144). Recommendations on parameters selection for the EHTM at DaRUS (10.18419/darus-1144). The EHTM muscle-specific parameters data for male and female arm muscles in the Appendix E. List of Muscle-Specific Parameters for Arm Muscles Used in the Study. The example set of the EHTM muscle-specific parameters data for female cervical muscles in the Appendix F. List of Muscle-Specific Parameters for the VIVA OpenHBM Neck Muscles and the whole set in the supplementary material.

## Appendix A Description of the model elements

The total MTU length is the internal Degree of Freedom (DOF) in the EHTM. It consists of the muscle fibre length $l_{CE}$ added to the tendon length $l_{SEE}$, represented in the EHTM model by the contractile and the serial elastic elements, respectively, see Fig. 4b:

A1 $$\begin{aligned} l_{MTU} = l_{CE} + l_{SEE}. \end{aligned}$$

At each point in time, the interacting forces from all four model elements are in equilibrium at the middle point *P*. The following equilibrium describes the equation of motion for this internal degree of freedom:

A2 $$\begin{aligned} \begin{aligned}&F_{CE}(l_{CE},\dot{l}_{CE},q) + F_{PEE}(l_{CE}) \\&\quad= F_{SEE}(l_{MTU},l_{CE}) + F_{SDE}(l_{CE},\dot{l}_{CE},\dot{l}_{MTU},q). \end{aligned} \end{aligned}$$

The contractile element force, $F_{CE}$ The force is generated by the active fibre bundles of the MTU during the muscle contraction and shows a different characteristic for the muscle shortening $\dot{l}_{CE} \le 0$ (concentric contraction) and lengthening $\dot{l}_{CE} > 0$ (eccentric contraction):

A3 $$\begin{aligned} \begin{aligned}&F_{CE,limb}(l_{CE},\dot{l}_{CE},q) \\&\quad= F_{max} \left( \frac{q \cdot F_{isom}+A_{rel,limb}}{1-\frac{\dot{l}_{CE}}{B_{rel,limb} \cdot l_{CE,opt}}} -A_{rel,limb}\right) , \end{aligned} \end{aligned}$$

where $l_{CE,opt}$—optimal length of the muscle fibres at which the maximal isometric force is generated; $\dot{l}_{CE}$—fibre contraction velocity; *q*—muscle activity level with the boundaries $q_{0} \le q \le 1$; $F_{max}$—maximum isometric force. The normalized Hill parameters $A_{rel,limb}$ and $B_{rel,limb}$ define the shape of the force-velocity relation curve for concentric ($A_{rel,con}; B_{rel,con}$) and eccentric ($A_{rel,ecc}; B_{rel,ecc}$) limbs serving as hyperbolas asymptotes as shown in Fig. 16a are calculated using formulas provided in (Haeufle et al 2014; Kleinbach et al 2017) and are used in the EHTM input parameters as constants. The isometric force-length relation is defined as:

A4 $$\begin{aligned} F_{isom}(l_{CE}) = \exp \left( - \left| \frac{\frac{l_{CE}}{l_{CE,opt}}-1}{\Delta W_{limb}} \right| ^{\nu _{CE,limb}} \right) , \end{aligned}$$

where $\Delta W_{limb}$ is a width of the normalized bell curve depicted in Fig. 16b and $\nu _{CE,limb}$ its exponent. Theses parameters could be chosen individually for ascending and descending limbs. Additional examples of the different force-length relations for the CE are provided in (Bayer et al 2017) in Fig.3.

The parallel elastic element force, $F_{PEE}$ The force originates from the elasticity of the connective tissues which surround the muscle belly and the muscle fibres which protect CE from overstretching. It is generated when the $l_{CE}$ length is equal to or greater than the passive element rest length $l_{PEE,0}=\mathcal {L}_{PEE,0} \cdot l_{CE,opt}$, see Fig. 16b:

A5 $$\begin{aligned} \begin{aligned}&F_{PEE}(l_{CE})= \left\{ \begin{array}{ll} 0, &{} l_{CE}<l_{PEE,0}\\ K_{PEE}(l_{CE,opt}-l_{PEE,0})^{\nu _{PEE}} &{} l_{CE} \ge l_{PEE,0}\end{array}\right. , \end{aligned} \end{aligned}$$

where coefficient

A6 $$\begin{aligned} \begin{aligned}&K_{PEE} = \mathcal {F}_{PEE}\frac{F_{max}}{(l_{CE,opt}(\Delta W_{desc}+1-\mathcal {L}_{PEE,0}))^{\nu _{PEE}}}, \end{aligned} \end{aligned}$$

depends on the parameters chosen by the user: $\mathcal {F}_{PEE}$—linear scaling factor of $F_{PEE}$, $\mathcal {L}_{PEE,0}$—rest length of PEE normalised to $l_{CE,opt}$ and $\nu _{PEE}$—exponent of $F_{PEE}$. They are defined by the force-length relation of modelled muscle (Fig. 16b).

The serial elastic element force, $F_{SEE}$ The nature of the force is similar to $F_{PEE}$ but for the tendon tissues. It is modelled as a nonlinear toe-region with a linear continuation:

A7 $$\begin{aligned} \begin{aligned}&F_{SEE}(l_{MTU},l_{CE}) = \\&\left\{ \begin{array}{ll} 0, &{} l_{SEE}<l_{SEE,0}\\ K_{SEE,nl} \cdot (l_{SEE}-l_{SEE,0})^{\nu _{SEE}}, &{} l_{SEE}<l_{SEE,nll}\\ \Delta F_{SEE,0} + K_{SEE,l}(l_{SEE}-l_{SEE,nll}), &{} l_{SEE} \ge l_{SEE,nll}\end{array}\right.  \end{aligned} \end{aligned}$$

Eq. A7 implies that there is no force generated until the tendon resting length $l_{SEE,0}$ is reached by the tendon length $l_{SEE}$, which is derived from Eq. A1. Afterwards, the force is defined by the power-law depending on $\nu _{SEE}$ between $l_{SEE,0}$ and the nonlinear–linear transition zone length $l_{SEE,nll}$ and linear progression for further extension after it. All curve parameters here can be derived from the tendon rest length—$l_{SEE,0}$, the relative stretch at nonlinear–linear transition—$\Delta U_{SEE,nll}$, the force at the nonlinear–linear transition—$\Delta F_{SEE,0}$ and the relative additional stretch in the linear part—$\Delta U_{SEE,l}$ using:

A8 $$\begin{aligned} l_{SEE,nll}&=l_{SEE,0}(1+ \Delta U_{SEE,nll}),\nonumber \\ \textstyle{\nu}_{SEE} \quad &= \Delta U_{SEE,nll}/\Delta U_{SEE,l},\hfill \\ K_{SEE,nl}&= \Delta F_{SEE,0}/(\Delta U_{SEE,nll} \cdot l_{SEE,0}){^{\nu_ {SEE}}},\nonumber \\ K_{SEE,l} \,\, &= \Delta F_{SEE,0}/(\Delta U_{SEE,l} \cdot l_{SEE,0}). \end{aligned}$$

The serial damping element force, $F_{SDE}$ This force represents the force-dependent damping behaviour of the tendon and is calculated as follows:

A9 $$\begin{aligned} \begin{aligned}&F_{SDE}(l_{CE},\dot{l}_{CE},\dot{l}_{MTU},q) \\&\quad= d_{SDE,max} \cdot \dot{l}_{SDE} \cdot \left( (1{-}R_{SDE}) \frac{F_{CE}{+}F_{PEE}}{F_{max}}{+}R_{SDE} \right) , \end{aligned} \end{aligned}$$

where $R_{SDE} \le 1$ – damping value at $F_{MTU}~=~0$; $\dot{l}_{SDE}=\dot{l}_{MTU} - \dot{l}_{CE}$—contraction velocity of the SDE; $d_{SDE,max}$—the maximum damping coefficient at $F_{MTU} = F_{max}$ calculated as

A10 $$\begin{aligned} d_{SDE,max}=D_{SDE} \frac{F_{max} \cdot A_{rel,0}}{l_{CE,opt} \cdot B_{rel,0}}. \end{aligned}$$

## Appendix B Contraction dynamics

To solve the force equilibrium in Eq. A2, the contraction velocity $\dot{l}_{CE}$ is determined from the quadratic differential equation (Günther et al 2007; Mörl et al 2012), taking into account the following equation for coefficients $C_{0,{limb}} < 0$ and $C_{1,{limb}} < 0$:

B11 $$\begin{aligned} C_{2,{limb}} \cdot \dot{l}_{CE}^2+C_{1,{limb}} \cdot \dot{l}_{CE}+C_{0,{limb}}\ = 0. \end{aligned}$$

Two different solutions for $\dot{l}_{CE}$ exist for concentric and eccentric cases:

B12 $$\begin{aligned} \dot{l}_{CE}=\left\{ \begin{array}{ll} \frac{-C_{1,{con}}-\sqrt{C_{1,{con}}^2-4 \cdot C_{2,{con}} \cdot C_{0,{con}}}}{2 \cdot C_{2,{con}}}\,, &{}\quad \dot{l}_{CE} \le 0,\\ \frac{-C_{1,{ecc}}+\sqrt{C_{1,{ecc}}^2-4 \cdot C_{2,{ecc}} \cdot C_{0,{ecc}}}}{2 \cdot C_{2,{ecc}}}\,, &{}\quad \dot{l}_{CE} > 0, \end{array}\right. \end{aligned}$$

with the appropriate coefficient values calculated using non-specific muscle parameters according to (Haeufle et al 2014; Kleinbach et al 2017):

B13 $$\begin{aligned} \begin{aligned}D_{0,{limb}}&=l_{CE,opt} \cdot B_{rel,limb} \cdot d_{SDE,max} \\&\quad \cdot \left( R_{SDE}+\left( 1-R_{SDE}\right) \left( q \cdot F_{isom}+\frac{F_{PEE}}{F_{max}}\right) \right) , \\C_{0,{limb}}&=D_{0,{limb}} \cdot \dot{l}_{MTU} + l_{CE,opt} \cdot B_{rel,limb} \\&\quad \cdot \left( F_{SEE} - F_{PEE}-F_{max} \cdot q \cdot F_{isom}\right) , \\C_{1,{limb}}&=-C_{2,{limb}} \cdot \dot{l}_{MTU}-D_0-F_{SEE} \\&\quad + F_{PEE}-F_{max} \cdot A_{rel,limb}, \\C_{2,{limb}}&=d_{SDE,max} \\&\quad \cdot \left( R_{SDE}-\left( A_{rel,limb}-\frac{F_{PEE}}{F_{max}}\right) \left( 1-R_{SDE}\right) \right) . \end{aligned} \end{aligned}$$

## Appendix C Activation dynamics

The force generation in a muscle is a transient process that starts with an excitation signal coming from the nervous system and ends with a building-up of cross-bridges caused by an increase in the concentration of calcium ions Ca${}^{2+}$. In the same way, muscle relaxation initiates with the dissipation of the Ca${}^{2+}$ ions, usually occurring much slower than the augmentation. This process is called the *activation dynamics* of the muscle. Mathematically it can be described with a first-order differential equation formulated in several ways. The EHTM provides three different formulations for the activation dynamics.

The first notation was proposed by Zajac (Zajac 1989) and amended with minimum muscle activity level $q_0$ by (Günther et al 2007):

C14 $$\begin{aligned} \begin{aligned}&\dot{q} (STIM)\\ &\quad= \frac{1}{\tau _{act}} \cdot \Bigl ( STIM - STIM\left( 1-\beta _{q}\right) \left( q-q_0\right) - \beta _{q}\left( q-q_0\right) \Bigr ), \end{aligned} \end{aligned}$$

where *q*—muscle activity level; $q_0$—minimum of the muscle activity level, which reflects the fact that some cross-bridges in real MTU will always be activated despite having no excitation signal; $\tau _{act}$—time constant of rising activation; *STIM*—muscle stimulation; $\beta _{q}$—ratio time constant of rising and falling activation, which is usually set as $\beta _{q}<1$ taking into account slower $Ca{}^{2+}$ ions dissipation in a muscle.

The second formulation of the activation dynamics was proposed by Hatze (Hatze 1977):

C15 $$\begin{aligned} q (\gamma _{rel}) = \frac{q_0+(\rho \cdot \gamma _{rel})^3}{1+(\rho \cdot \gamma _{rel})^3}. \end{aligned}$$

In addition to the differential equation for $Ca{}^{2+}$ ions concentration

C16 $$\begin{aligned} \begin{aligned}&\dot{\gamma }_{rel} (STIM) = m(STIM-\gamma _{rel}), \\&with \quad \gamma _{rel}(t_0)=0, \end{aligned} \end{aligned}$$

it has an algebraic equation which depicts the length-dependent $Ca{}^{2+}$ ions sensitivity of the muscle: The longer a muscle is, the more it is activated with the same stimulation.

C17 $$\begin{aligned} \rho (l_{CE,rel}) = c \cdot \eta \frac{(k-1)}{(k-l_{CE,rel})}l_{CE,rel}, \end{aligned}$$

where $l_{CE,rel} = l_{CE} / l_{CE,opt}$—relative length of CE; *m*, *k*, *c* and $\eta$—Hatze constants.

The third formulation available in the EHTM is an updated version of the Hatze activation dynamics (Hatze 1977) proposed by (Rockenfeller and Günther 2018) in which the equation describing the $Ca{}^{2+}$ ions sensitivity of the muscle was simplified to:

C18 $$\begin{aligned} \rho (l_{CE,rel}) = c \cdot \eta \cdot l_{CE,rel}, \end{aligned}$$

where $l_{CE,rel} = l_{CE} / l_{CE,opt}$—relative length of the CE; *m*, *c* and $\eta$—Hatze constants. It results in faster code execution with similar calculated values for the muscle activity.

The main difference in the resulting muscle activity level over time when using Zajac and Hatze activation dynamics formulations is illustrated in Fig. 17. One can see muscle activity curves given for a range of muscle stimulation levels *STIM* from 0.1 to 1.0 with recommended values for the constants presented in Table 1. The Hatze activation dynamics provides a more biofidelic response resulting in a slighter decrease in muscle activity level over time due to its higher nonlinear dependency over STIM. Based on the studies (Rockenfeller and Günther 2016, 2018), it is recommended to use the updated version of activation dynamics proposed by Hatze (formulation three) for better performance when running simulations with the EHTM.

## Appendix D Detailed description of model and simulation set-ups used in the study

**Table 3:** Comparison of the Finite Element Human Body Models used in the study

Model Name	THUMS v5	VIVA OpenHBM
Published in	(Kato et al 2017)	(Östh et al 2017a)
Population represented	50th percentile male	50th percentile female
Total number of elements	285,550	318,400
Active muscle elements	Whole body	Neck region only
Muscle controller	PID for the whole body	Absent
Appearance		

### D.1 Emergency braking

Model set-up: The THUMS v5 occupant model was used for the emergency braking load case verification. The simulations were performed with both the original THUMS v5 model and a modified THUMS v5-EHTM version, in which the muscle modelling was adjusted to accommodate the use of the EHTM material (Martynenko et al 2020). The muscles in the original THUMS v5 are modelled using truss elements with the *MAT_MUSCLE material assigned, while tendons are represented by seatbelt elements. In contrast to *MAT_MUSCLE, the EHTM material has the advantage of including tendon as an element of the Hill-type muscle model itself, see Fig. 4b. Therefore, muscles formerly modelled as a combination of muscle and seatbelt elements were replaced by truss elements using only the EHTM material. This process eliminated the need for tendon routing via slip rings, as the EHTM material allows for routing with the *PART_AVERAGED keyword. Shell elements representing tendons with broad contact areas to adjacent bones were kept intact, as the required joint stability cannot be achieved by using only EHTM material truss elements. The material parameters of *MAT_MUSCLE were converted to the corresponding parameters of the EHTM material with missing data added from anatomic literature where necessary. A detailed description of the conversion method can be found in the supplementary materials (Nölle et al 2022) or appendices of (Wochner et al 2022).

Simulation set-up: The experimental data used to verify the passive material properties of the EHTM were collected as part of the OM4IS project to create an extensive collection of combined vehicle and occupant kinematic data based on human volunteer driving experiments covering a range of different manoeuvrers (Huber et al 2015). The simulation set-up used to compare the passive elastic and damping behaviour of *MAT_MUSCLE with the EHTM was set to mimic the experimental conditions during the volunteer 50 km/h emergency braking trials. The THUMS v5 AHBM was placed in the front passenger seat and secured with a three-point seatbelt. The hands were held in front of the body, while the feet were placed on a footrest. Generic models of the seat, seatbelt and the vehicle interior used for all simulations are described in the technical report “Deliverable 2.2 of the OSCCAR H2020 project” (Leitgeb et al 2021). All interior parts combined formed a sled with which the AHBM velocity can be controlled. The sled model was first accelerated to a constant velocity of 50 km/h, after which a breaking pulse was applied to achieve a complete stop. Two simulations, one for each muscle material, were performed. All the muscles were completely relaxed during the simulations with a minimum physiological activity level set to $q_{0}=0.005$ (Günther 1997). The displacement of the head’s COG in the sagittal plane was tracked throughout the entire simulation runtime and compared to the corresponding experimental results.

### D.2 Fast elbow joint movements

Model set-up: Upper extremities were derived from the THUMS v3 (Iwamoto et al 2007) and the VIVA OpenHBM (Östh et al 2017a) models and enhanced by including nine EHTM muscle elements. The soft tissues were removed, and their appropriate inertia properties were applied to the bone segments, which were made rigid, resulting in a rigid body musculoskeletal system. In addition, each arm model was repositioned and adapted to match the experimental setup from (Kistemaker et al 2006): shoulder bones were fixed in space, a rigid connection between the shoulder and the upper arm was set and an idealised revolute joint representing the elbow joint was added. In the THUMS v3 arm model, the forearm bones were also merged with the hand bones to define a single segment. The unspecific default muscle parameters for the EHTM material given in Table 1 were applied. To define the muscle-specific parameters, first, the optimal muscle fibre length $l_{CE,opt}$ of the VIVA OpenHBM arm model was chosen based on parameters presented in (Holzbaur et al 2005; Murray et al 2000), scaled to the female parameters by the length ratio of radius bones between the 50th percentile male THUMS v3 and the 50th percentile female VIVA OpenHBM model. Second, the tendon slack length $l_{SEE,0}$ was determined such that the VIVA OpenHBM arm model matched the maximum isometric torque vs angular displacement curve, published in (Holzbaur et al 2007) with its peak torque at 90${}^\circ$ flexion angle for both, flexor and extensor muscles. For the THUMS v3 arm, both optimal fibre length and tendon slack length were adapted and scaled by the individual muscle-tendon complex length change from the VIVA OpenHBM to the THUMS v3 model, where $l_{MTU,opt}=l_{CE,opt}+l_{SEE,0}$. Third, the maximum force capacity values $F_{max}$ of each individual muscle were chosen based on the peak force values in  (Holzbaur et al 2007) and scaled as a functional group (flexor and extensor muscles). The scaling factors for extensors and flexors were chosen separately to fit the experimental corridor of isometric torque. For the female VIVA OpenHBM  arm model, the torque reference corridor was additionally scaled with data from  (Buchanan et al 1998), since the torque progression values in  (Holzbaur et al 2007) corresponded exclusively to male subjects. The final muscle parameters for both models are provided in Appendix E, Tables 5 and 6. Four of the nine implemented muscles (*triceps short, medial and long head, biceps long head*) were routed at nodes fixed to the corresponding bone part using *PART_AVERAGED keyword.

Simulation set-up: The fast forearm movements between elbow joint angles of 45${}^\circ$, 95${}^\circ$ and 145${}^\circ$ reported in (Kistemaker et al 2006) were simulated with the upper extremity models after prior optimisation. To decrease the manifold of possible solutions and thus make the optimisation process more efficient, the muscles were combined into six groups, see Appendix E. During the optimisation process, multiple simulations were performed to reach desired elbow angles by varying $STIM_{\alpha }$ values. In the found equilibrium position, an external perturbation was applied to the VIVA OpenHBM arm model to evaluate the position robustness against the external force (low-frequency joint stiffness) as denoted above in Eq. 13. After a suitable set of stimulation values was found, the corresponding muscle fibre lengths $l_{CE}$ in the equilibrium position were used as feedback-controller target values for the $\lambda$– and hybrid controller. The $\lambda$ target values were scaled by the muscle-tendon complex length difference ratio and transferred to the THUMS v3 arm model, followed by three iterations of simulations with the target values for a hybrid controller. The final muscle stimulation values $STIM_{i,hyb}(t)$ of each equilibrium position were applied as open-loop stimulation values $STIM_{i,\alpha }(t)$ for the hybrid controller in the next iteration. This way, new open-loop and closed-loop controller target values for the stronger muscles (representing male subject) of the THUMS v3 arm model could be determined without further optimisation. The $\alpha$–, $\lambda$– and hybrid controllers were used in separate simulations to evaluate their performance compared to the original experimental data from (Kistemaker et al 2006).

**Table 4:** Comparison of the upper extremity finite element models used in the study

Model Name	THUMS v3	VIVA OpenHBM
Published in	(Iwamoto et al 2007)	(Östh et al 2017a)
Rigid bodies	3	3
Degrees of freedom	4	4
Number of muscles	9	9
Appearance		

### D.3 Stretch reflex of the cervical muscles

Model set-up: The functionality of the muscle stretch reflex controller implemented in the EHTM was verified using the VIVA OpenHBM model (Östh et al 2017a). This 50th percentile average female Open-Source Human Body Model has some simplifications like rigid bones and kinematic joints but includes fully deformable soft tissues and a detailed neck with 34 cervical muscles. They are represented by 252 elements, 56 of which are routed, and modelled with the one-dimensional Hill-type muscle material *MAT_MUSCLE (Östh et al 2017b). General information on the VIVA OpenHBM is provided in Table 3, while neck muscle elements are shown in Fig. 18.

For the following analysis, the existing material definition of all muscle elements in the neck region of the VIVA OpenHBM was replaced with the EHTM. Material parameters from *MAT_MUSCLE could not be applied directly for the EHTM due to missing information of tendon lengths. Therefore, to obtain a suitable set of parameters for the EHTM following procedure was performed:

Firstly, physiological cross-sectional area (*PCSA*) values for cervical muscles were obtained from (Borst et al 2011) and scaled using coefficients which accounted for the fact that initial values correspond to a 86 years old 50th percentile embalmed male subject (Kleinbach 2019). In addition, to translate these values to a 25 years old 50th percentile female model, they were multiplied by a factor of 1.64, leading to a middle-aged male (Van Ee et al 2000), followed by a division through a factor of 1.70 (Marras et al 2001; Östh et al 2017b) leading to middle-aged woman. The needed maximum isometric force $F_{max}$ parameter for each muscle could be found by multiplying the maximum isometric stress by the muscle cross-sectional area *PCSA*:

D19 $$\begin{aligned} F_{max} = \sigma _{isom,max} \cdot PCSA, \end{aligned}$$

where the maximum isometric stress lies in the interval 0.2 MPa $< \sigma _{isom,max}<$ 1.0 MPa (Winters and Stark 1988; Östh et al 2017b). The value $\sigma _{isom,max}=0.5$ MPa used in current contribution was determined by comparing simulations of isometric contraction experiments for all muscles modelled with *MAT_MUSCLE and the EHTM.

Secondly, biologically adequate values for the remaining two muscle-specific parameters $l_{CE,opt}$ and $l_{SEE,0}$ had to be found. One needs to solve the problem of geometrical nonconformity between muscle and tendon lengths given in the experimental data set (Borst et al 2011) and the modelled musculoskeletal system in the VIVA OpenHBM. This problem arises because of several simplifications and assumptions made during the model creation. The VIVA OpenHBM model authors neglected tendon lengths for muscle fascicles in the model, arguing that data are only available for some of them from the experiment, and compensated it by change of generic muscle parameters (Östh et al 2017a). Such an approach is unsuitable for the EHTM as it requires explicit definition of resting tendon length $l_{SEE,0}$ and optimal muscle fibre length $l_{CE,opt}$. These values were determined under the assumption that in the initial relaxed state of the muscle its length is equal to the sum of both values (Günther et al 2007). Considering the state of the VIVA OpenHBM model at the start of the simulation as “relaxed”, an empirical algorithm shown in Fig. 19 could be proposed. Based on the experimental measurements, the tendon length is either scaled to the muscle element length in the model, if a value is provided, or its length is set to a default small value of $l_{SEE,0}=0.001$ m in the absence of data. The calculation of $l_{CE,opt}$ is based on the total muscle-tendon unit length existing in the VIVA OpenHBM. The obtained values are given in Table 7 from Appendix F. To verify the validity and comparability of the parameters retrieved for the EHTM with the *MAT_MUSCLE, the original test load case from (Östh et al 2017b) was simulated. The flexor and extensor muscles with the EHTM material were activated with the same stimulation values as for the original one. Afterwards, the maximum isometric force produced by the head–neck model in each direction was compared for both materials. Verification and validation process is described in details in (Kleinbach 2019). More details on the parameters selection procedure for the EHTM material can be found in the supplementary materials to this manuscript (Nölle et al 2022).

Simulation set-up: The “Falling Heads” experimental data from (Wochner et al 2022) were used to verify the insertion of the EHTM material to the VIVA OpenHBM with the experiment scheme shown in Fig. 20. The idea of the experiment was to investigate reflex-based muscle activation under gravitational load in a simple environment with explicit initial conditions, which would be easy to simulate. Such a loading type, when related to the automotive context, can be compared to a slight rear impact which fits perfectly in a research scope for the VIVA OpenHBM (Östh et al 2017b). Ten female volunteers were asked to lie down on their back on an experimental table with the head supported by a specifically designed trapdoor device. The table height and position in relation to the trapdoor were controlled to account for the difference in volunteer’s anthropometry. Surface electromyography (EMG) was used to monitor the thorough relaxation of the cervical muscles before the start of the experiment. After a specific time, unknown to volunteers, the trapdoor device was released suddenly, causing a downward head movement under gravity. Head kinematics were recorded with a high-speed camera followed by tracking of markers with special software, resulting in a corridor of measured coordinates for the head centre of gravity (COG).

Prior to the simulations, the model was repositioned in space from its standard sitting position to a supine position to match the experiment’s volunteer posture on the table as seen in Fig. 21(a). Afterwards, the settling simulation with gravity loading was performed. The parts of the VIVA OpenHBM below the T5 vertebra were fixed, contacts to a rigid table with a trapdoor set and runtime determined to let the model settle down until complete relaxation as shown in Fig. 21(b). Finally, a trapdoor was released, leading to the fall of the head with subsequent automatic activation of the reflex controller, see Fig. 21(c).

## Appendix E List of muscle-specific parameters for arm muscles used in the study

The EHTM muscle-specific parameters $F_ {max}$, $l_ {CE, opt}$ and $l_ {SEE, 0}$ for upper extremity muscles are given below for the unit system mm–ms–kg–KN in Table 5 for the VIVA OpenHBM (Östh et al 2017a) and in Table 6 for the THUMS v3 (Iwamoto et al 2007) models. In addition, muscle names, the unique part identification number in LS-DYNA and a functional group (flexor or extensor) are specified.

**Table 5:** Muscle-specific parameters of the VIVA OpenHBM model

Muscle name	id	Group	$F_{max}$[kN]	$l_{CE,opt}$[mm]	$l_{SEE,0}$[mm]
Biceps long head	BILH	BEF	0.283	109.18	193.62
Biceps short head	BISH	BEF	0.195	124.24	135.66
Brachialis	BRAC	–	0.447	80.94	66.47
Brachioradialis	BRAD	–	0.120	162.82	108.36
Triceps long head	TRLO	–	0.556	96.00	204.04
Triceps lateral head	TRLA	MEE	0.439	79.06	170.19
Triceps medial head	TRME	MEE	0.439	59.29	104.69
Pronator teres	PRTE	MEF	0.252	46.12	43.56
Extensor carpi rad. long	ECRL	MEF	0.139	76.24	187.14

**Table 6:** Muscle-specific parameters of the THUMS v3 model

Muscle name	id	Group	$F_{max}$[kN]	$l_{CE,opt}$[mm]	$l_{SEE,0}$[mm]
Biceps long head	BILH	BEF	0.624	119.30	211.58
Biceps short head	BISH	BEF	0.436	138.57	151.30
Brachialis	BRAC	–	0.987	85.85	70.50
Brachioradialis	BRAD	–	0.261	180.59	120.18
Triceps long head	TRLO	–	0.799	105.62	203.93
Triceps lateral head	TRLA	MEE	0.624	84.64	182.20
Triceps medial head	TRME	MEE	0.624	65.08	114.91
Pronator teres	PRTE	MEF	0.566	51.97	49.09
Extensor carpi rad. long	ECRL	MEF	0.305	83.08	203.93

## Appendix F List of muscle-specific parameters for the VIVA OpenHBM neck muscles

Example data for the EHTM muscle-specific parameters $F_ {max}$, $l_ {CE, opt}$ and $l_ {SEE, 0}$ are given in Table 7 for the unit system mm–ms–kg–KN. These data are extracted from the VIVA OpenHBM version 20161202, where 252 muscle fascicles are implemented. Muscle name and the unique part identification number in LS-DYNA (*PID) correspond to the already existing in the model. The whole list is available in the supplementary material to the current manuscript. Detailed explanation to parameters derivation is given in Sect. D.3 Stretch Reflex of the Cervical Muscles. Each muscle fascicle is assigned a number for easier orientation, which could be addressed instead of the long, complicated name in the text.

**Table 7:** Specific parameters for the cervical spine muscles implemented in the VIVA OpenHBM

Nr	Muscle name	PID	$F_{max}$ [kN]	$l_{CE,opt}$ [mm]	$l_{SEE,0}$ [mm]
1	N_M_R3C4_L_Iliocostalis_Cervicis	2008501	0.0071	80.863	23.507
2	N_M_R3C5_L_Iliocostalis_Cervicis	2008502	0.0071	79.915	11.151
3	N_M_R3C6_L_Iliocostalis_Cervicis	2008503	0.0071	74.533	1.000
$\cdots$	$\cdots$	$\cdots$	$\cdots$	$\cdots$	$\cdots$
$\cdots$	$\cdots$	$\cdots$	$\cdots$	$\cdots$	$\cdots$
250	N_M_SClav_R_Trap_Desc	2008127	0.03835	235.309	7.715
251	N_M_C7Scap_R_Trap_Trans	2008128	0.1285	72.874	94.836
252	N_M_C6Scap_R_Trap_Trans	2008129	0.119	66.991	97.910
