# Supplementary material for: Development and verification of a physiologically motivated internal controller for the open-source extended Hill-type muscle model in LS-DYNA
Source: Biomech Model Mechanobiol. 2023 Aug 5;22(6):2003–32. doi: 10.1007/s10237-023-01748-9 (PMC10613192; doi:10.1007/s10237-023-01748-9)
Supplement: Supplementary file 1 — (pdf 108 KB) [file 10237_2023_1748_MOESM1_ESM.pdf]

List of the Extended Hill-Type Muscle Material Model Muscle Specific Parameters for the VIVA OpenHBM Cervical Muscles

This document is a supplementary material to the publication ”Development and Validation of a Physiologically Motivated Internal Controller for the Open-Source Extended Hill-type Muscle Model in LS-DYNA”. It lists the open-source Extended Hill-Type Muscle (EHTM) material model muscle specific parameters F_{max}, $l_{CE,opt}$ and $l_{SEE,0}$ for all of 252 muscle fascicles implemented in the VIVA OpenHBM version 20161202. Parameters are given in the Table 1 below for the unit system mm–ms–kg–KN. Muscle name and the unique part identification number in LS-DYNA (*PID) corresponds to the already existing in the model. Detailed explanation to parameters derivation is given in the manuscript's Section 2.5.3 ”Stretch Reflex of The Cervical Muscles”. Each muscle fascicle is assigned a number for easier orientation, which could be addressed instead of the long, complicated name in the text.

Table 1: The EHTM muscle specific parameters for the VIVA OpenHBM cervical muscles.

Nr.	Muscle names	PID	$\mathbf{F_{max}}$ [kN]	$\mathbf{l_{CE,opt}}$ [mm]	$\mathbf{l_{SEE,0}}$ [mm]
1	N_M_R3C4_L_Iliocostalis_Cervicis	2008501	0.0071	80.863	23.507
2	N_M_R3C5_L_Iliocostalis_Cervicis	2008502	0.0071	79.915	11.151
3	N_M_R3C6_L_Iliocostalis_Cervicis	2008503	0.0071	74.533	1.000
4	N_M_T2S_L_Longissimus_Capitis	2008504	0.00625	112.918	16.131
5	N_M_T1S_L_Longissimus_Capitis	2008505	0.00625	89.111	16.549
6	N_M_C7S_L_Longissimus_Capitis	2008506	0.00625	72.934	28.132
7	N_M_C6S_L_Longissimus_Capitis	2008507	0.00625	62.858	26.939
8	N_M_C5S_L_Longissimus_Capitis	2008508	0.00625	52.871	24.170
9	N_M_C4S_L_Longissimus_Capitis	2008509	0.00625	47.730	17.047
10	N_M_T2C1_L_Longissimus_Cervicis	2008510	0.00775	74.937	43.794
11	N_M_T2C2_L_Longissimus_Cervicis	2008511	0.00775	65.359	33.953
12	N_M_T2C3_L_Longissimus_Cervicis	2008512	0.00775	62.655	28.480
13	N_M_T2C4_L_Longissimus_Cervicis	2008513	0.00775	52.835	20.585
14	N_M_T2C5_L_Longissimus_Cervicis	2008514	0.00775	46.586	15.125
15	N_M_T2C6_L_Longissimus_Cervicis	2008515	0.00775	38.846	10.090
16	N_M_R3C2_L_Longissimus_Cervicis	2008516	0.0164	72.677	53.881
17	N_M_R3C3_L_Longissimus_Cervicis	2008517	0.0164	70.638	46.280
18	N_M_C6C7_L_Interspinalis_Cervicis	2008519	0.0061	15.390	1.000
19	N_M_C5C6_L_Interspinalis_Cervicis	2008520	0.0128	9.769	1.000
20	N_M_C4C5_L_Interspinalis_Cervicis	2008521	0.0081	12.807	1.000
21	N_M_C3C4_L_Interspinalis_Cervicis	2008522	0.00855	20.791	1.000
22	N_M_C2C3_L_Interspinalis_Cervicis	2008523	0.0049	10.994	1.000
23	N_M_C1C2_L_InTrans_Anterior_Cervicis	2008524	0.00405	15.055	1.000
24	N_M_C2C3_L_InTrans_Anterior_Cervicis	2008525	0.0111	15.226	1.000
25	N_M_C3C4_L_InTrans_Anterior_Cervicis	2008526	0.011	12.740	1.000
26	N_M_C4C5_L_InTrans_Anterior_Cervicis	2008527	0.01075	16.860	1.000
27	N_M_C5C6_L_InTrans_Anterior_Cervicis	2008528	0.0082	16.380	1.000
28	N_M_C6C7_L_InTrans_Anterior_Cervicis	2008529	0.00755	17.893	1.000
29	N_M_C1C2_L_InTrans_Posterior_Cervicis	2008530	0.00805	22.126	1.000
30	N_M_C2C3_L_InTrans_Posterior_Cervicis	2008531	0.00855	9.432	1.000
31	N_M_C3C4_L_InTrans_Posterior_Cervicis	2008532	0.0076	17.620	1.000
32	N_M_C4C5_L_InTrans_Posterior_Cervicis	2008533	0.00775	13.644	1.000
33	N_M_C5C6_L_InTrans_Posterior_Cervicis	2008534	0.0095	13.823	1.000
34	N_M_C6C7_L_InTrans_Posterior_Cervicis	2008535	0.01175	15.452	1.000
35	N_M_C1Scap_L_Lev_Scapulae	2008536	0.0509	161.225	8.298
36	N_M_C2Scap_L_Lev_Scapulae	2008537	0.0299	127.666	22.157
37	N_M_C3Scap_L_Lev_Scapulae	2008538	0.02155	112.242	29.537
38	N_M_C4Scap_L_Lev_Scapulae	2008539	0.0198	101.543	27.078
39	N_M_SC6_L_Longus_Capitis	2008540	0.01115	89.327	1.000
40	N_M_SC5_L_Longus_Capitis	2008541	0.01115	78.348	1.000
41	N_M_SC4_L_Longus_Capitis	2008542	0.01115	66.256	1.000
42	N_M_SC3_L_Longus_Capitis	2008543	0.01115	51.863	1.000
43	N_M_C4C1_L_Longus_Colli	2008544	0.00615	44.046	1.000
44	N_M_C4C2_L_Longus_Colli	2008545	0.00265	29.202	1.000
45	N_M_C7C5_L_Longus_Colli	2008546	0.00765	41.334	1.000
	Continued on next page				

Table 1 – Continued from previous page

Nr.	Muscle names	PID	$\mathbf{F_{max}}$ [kN]	$\mathbf{l_{CE,opt}}$ [mm]	$\mathbf{l_{SEE,0}}$ [mm]
46	N_M_C7C4_L_Longus_Colli	2008547	0.00765	56.638	1.000
47	N_M_T1C3_L_Longus_Colli	2008548	0.00765	92.116	1.000
48	N_M_T1C2_L_Longus_Colli	2008549	0.00765	104.913	1.000
49	N_M_T1C1_L_Longus_Colli	2008550	0.00765	121.020	1.000
50	N_M_T4C7_L_Multifidus_Cervicis	2008551	0.01745	67.717	9.850
51	N_M_T3C7_L_Multifidus_Cervicis	2008552	0.01945	55.356	1.000
52	N_M_T3C6_L_Multifidus_Cervicis	2008553	0.01945	70.453	1.000
53	N_M_T2C6_L_Multifidus_Cervicis	2008554	0.0347	48.427	1.000
54	N_M_T1C5_L_Multifidus_Cervicis	2008555	0.01895	35.329	1.000
55	N_M_T1C4_L_Multifidus_Cervicis	2008556	0.02915	47.164	1.000
56	N_M_C7C3_L_Multifidus_Cervicis	2008557	0.028	56.346	1.000
57	N_M_C6C3_L_Multifidus_Cervicis	2008558	0.01805	43.066	1.000
58	N_M_C6C2_L_Multifidus_Cervicis	2008559	0.01805	55.815	1.000
59	N_M_C5C2_L_Multifidus_Cervicis	2008560	0.01805	44.573	1.000
60	N_M_C2C1_L_Obliquus_Cap_Inf	2008561	0.08565	45.241	1.000
61	N_M_C1S_L_Obliquus_Cap_Sup	2008562	0.0461	21.878	1.000
62	N_M_ScapH_L_Omohyoid	2008563	0.0222	239.111	1.000
63	N_M_SternH_L_SternoHyoid	2008565	0.0128	141.275	1.000
64	N_M_ClavH_L_SternoHyoid	2008566	0.00425	113.976	1.000
65	N_M_R1H_L_Sternothyroid	2008567	0.00985	130.961	1.000
66	N_M_SternH_L_Sternothyroid	2008568	0.01585	134.756	1.000
67	N_M_C1S_L_Rect_Cap_Ant	2008570	0.004	18.274	1.000
68	N_M_C1S_L_Rect_Cap_Lat	2008571	0.03915	17.222	1.000
69	N_M_C2S_L_Rect_Cap_Post_Maj	2008572	0.02705	42.721	1.000
70	N_M_C1S_L_Rect_Cap_Post_Min	2008573	0.04515	29.635	1.000
71	N_M_C6Scap_L_Rhomb_Min	2008574	0.0239	116.215	1.000
72	N_M_C7Scap_L_Rhomb_Min	2008575	0.0239	109.615	1.000
73	N_M_C4R1_L_Scalenus_Ant	2008576	0.01025	64.683	28.123
74	N_M_C5R1_L_Scalenus_Ant	2008577	0.01025	62.292	17.604
75	N_M_C6R1_L_Scalenus_Ant	2008578	0.02055	57.047	9.921
76	N_M_C1R1_L_Scalenus_Med	2008579	0.01315	65.012	60.678
77	N_M_C2R1_L_Scalenus_Med	2008580	0.01315	65.291	45.704
78	N_M_C3R1_L_Scalenus_Med	2008581	0.01315	73.325	28.108
79	N_M_C4R1_L_Scalenus_Med	2008582	0.01315	66.346	21.010
80	N_M_C5R1_L_Scalenus_Med	2008583	0.01315	59.972	14.993
81	N_M_C6R1_L_Scalenus_Med	2008584	0.01315	50.625	10.969
82	N_M_C7R1_L_Scalenus_Med	2008585	0.01315	49.628	3.309
83	N_M_C5R1_L_Scalenus_Post	2008586	0.0223	62.624	32.392
84	N_M_C6R1_L_Scalenus_Post	2008587	0.0223	61.149	24.249
85	N_M_T4S_L_Semispinalis_Capitis	2008588	0.023	199.272	1.000
86	N_M_T3S_L_Semispinalis_Capitis	2008589	0.0257	175.968	1.000
87	N_M_T2S_L_Semispinalis_Capitis	2008590	0.03145	150.655	1.000
88	N_M_T1S_L_Semispinalis_Capitis	2008591	0.0316	130.571	1.000
89	N_M_C7S_L_Semispinalis_Capitis	2008592	0.02095	120.197	1.000
90	N_M_C6S_L_Semispinalis_Capitis	2008593	0.0172	103.021	1.000
91	N_M_C5S_L_Semispinalis_Capitis	2008594	0.02285	92.347	1.000
92	N_M_C4S_L_Semispinalis_Capitis	2008595	0.02295	80.530	1.000
93	N_M_C3S_L_Semispinalis_Capitis	2008596	0.01765	68.575	1.000
94	N_M_T5C7_L_Semispinalis_Cervicis	2008597	0.0227	117.653	1.000
95	N_M_T5C6_L_Semispinalis_Cervicis	2008598	0.0227	131.975	1.000
96	N_M_T4C5_L_Semispinalis_Cervicis	2008599	0.0136	120.125	1.000
97	N_M_T4C4_L_Semispinalis_Cervicis	2008600	0.0136	131.917	1.000
98	N_M_T4C3_L_Semispinalis_Cervicis	2008601	0.0136	156.857	1.000
99	N_M_T3C4_L_Semispinalis_Cervicis	2008602	0.01765	117.199	1.000
100	N_M_T3C3_L_Semispinalis_Cervicis	2008603	0.01765	137.446	1.000
101	N_M_T3C2_L_Semispinalis_Cervicis	2008604	0.01765	145.575	1.000
102	N_M_T2C3_L_Semispinalis_Cervicis	2008605	0.0225	114.361	1.000
103	N_M_T2C2_L_Semispinalis_Cervicis	2008606	0.0225	126.073	1.000
104	N_M_T1R4_L_Serratus_Post_Sup	2008607	0.01265	41.544	58.321
105	N_M_C7R4_L_Serratus_Post_Sup	2008608	0.0251	53.975	52.145
106	N_M_C7R3_L_Serratus_Post_Sup	2008609	0.03295	49.302	42.399

Continued on next page

Table 1 – Continued from previous page

Nr.	Muscle names	PID	$\mathbf{F_{max}}$ [kN]	$\mathbf{l_{CE,opt}}$ [mm]	$\mathbf{l_{SEE,0}}$ [mm]
107	N_M_C6R1_L_Serratus_Post_Sup	2008610	0.028	24.503	29.403
108	N_M_C5S_L_Splenius_Capitis	2008611	0.0088	87.706	1.000
109	N_M_C6S_L_Splenius_Capitis	2008612	0.01395	103.460	1.000
110	N_M_C6S_L_Splenius_Capitis	2008613	0.0198	105.477	1.000
111	N_M_C7S_L_Splenius_Capitis	2008614	0.01935	121.936	1.000
112	N_M_C7S_L_Splenius_Capitis	2008615	0.01905	124.187	1.000
113	N_M_T1S_L_Splenius_Capitis	2008616	0.01935	140.981	1.000
114	N_M_T2S_L_Splenius_Capitis	2008617	0.0247	176.331	1.000
115	N_M_T3C1_L_Splenius_Cervicis	2008618	0.02475	124.921	56.337
116	N_M_T3C2_L_Splenius_Cervicis	2008619	0.02475	91.638	71.873
117	N_M_SternS_L_SCM1	2008620	0.0349	123.228	58.896
118	N_M_SternS_L_SCM2	2008621	0.062	137.172	41.914
119	N_M_ClavS_L_SCM1	2008622	0.02445	95.452	52.914
120	N_M_ClavS_L_SCM2	2008623	0.0237	115.480	33.489
121	N_M_C5Scap_L_Trap_Desc	2008624	0.05055	115.633	39.420
122	N_M_C5Scap_L_Trap_Desc	2008625	0.07115	128.325	40.955
123	N_M_C5Scap_L_Trap_Desc	2008626	0.0168	193.969	6.520
124	N_M_SClav_L_Trap_Desc	2008627	0.03835	235.309	7.715
125	N_M_C7Scap_L_Trap_Trans	2008628	0.1285	72.874	94.836
126	N_M_C6Scap_L_Trap_Trans	2008629	0.119	66.991	97.910
127	N_M_R3C4_R_Iliocostalis_Cervicis	2008001	0.0071	80.863	23.507
128	N_M_R3C5_R_Iliocostalis_Cervicis	2008002	0.0071	79.915	11.151
129	N_M_R3C6_R_Iliocostalis_Cervicis	2008003	0.0071	74.533	1.000
130	N_M_T2S_R_Longissimus_Capitis	2008004	0.00625	112.918	16.131
131	N_M_T1S_R_Longissimus_Capitis	2008005	0.00625	89.111	16.549
132	N_M_C7S_R_Longissimus_Capitis	2008006	0.00625	72.934	28.132
133	N_M_C6S_R_Longissimus_Capitis	2008007	0.00625	62.858	26.939
134	N_M_C5S_R_Longissimus_Capitis	2008008	0.00625	52.871	24.170
135	N_M_C4S_R_Longissimus_Capitis	2008009	0.00625	47.730	17.047
136	N_M_T2C1_R_Longissimus_Cervicis	2008010	0.00775	74.937	43.794
137	N_M_T2C2_R_Longissimus_Cervicis	2008011	0.00775	65.359	33.953
138	N_M_T2C3_R_Longissimus_Cervicis	2008012	0.00775	62.655	28.480
139	N_M_T2C4_R_Longissimus_Cervicis	2008013	0.00775	52.835	20.585
140	N_M_T2C5_R_Longissimus_Cervicis	2008014	0.00775	46.586	15.125
141	N_M_T2C6_R_Longissimus_Cervicis	2008015	0.00775	38.846	10.090
142	N_M_R3C2_R_Longissimus_Cervicis	2008016	0.0164	72.677	53.881
143	N_M_R3C3_R_Longissimus_Cervicis	2008017	0.0164	70.638	46.280
144	N_M_C6C7_R_Interspinalis_Cervicis	2008019	0.0061	15.390	1.000
145	N_M_C5C6_R_Interspinalis_Cervicis	2008020	0.0128	9.769	1.000
146	N_M_C4C5_R_Interspinalis_Cervicis	2008021	0.0081	12.807	1.000
147	N_M_C3C4_R_Interspinalis_Cervicis	2008022	0.00855	20.791	1.000
148	N_M_C2C3_R_Interspinalis_Cervicis	2008023	0.0049	10.994	1.000
149	N_M_C1C2_R_InTrans_Anterior_Cervicis	2008024	0.00405	15.055	1.000
150	N_M_C2C3_R_InTrans_Anterior_Cervicis	2008025	0.0111	15.226	1.000
151	N_M_C3C4_R_InTrans_Anterior_Cervicis	2008026	0.011	12.740	1.000
152	N_M_C4C5_R_InTrans_Anterior_Cervicis	2008027	0.01075	16.860	1.000
153	N_M_C5C6_R_InTrans_Anterior_Cervicis	2008028	0.0082	16.380	1.000
154	N_M_C6C7_R_InTrans_Anterior_Cervicis	2008029	0.00755	17.893	1.000
155	N_M_C1C2_R_InTrans_Posterior_Cervicis	2008030	0.00805	22.126	1.000
156	N_M_C2C3_R_InTrans_Posterior_Cervicis	2008031	0.00855	9.432	1.000
157	N_M_C3C4_R_InTrans_Posterior_Cervicis	2008032	0.0076	17.620	1.000
158	N_M_C4C5_R_InTrans_Posterior_Cervicis	2008033	0.00775	13.644	1.000
159	N_M_C5C6_R_InTrans_Posterior_Cervicis	2008034	0.0095	13.823	1.000
160	N_M_C6C7_R_InTrans_Posterior_Cervicis	2008035	0.01175	15.452	1.000
161	N_M_C1Scap_R_Lev_Scapulae	2008036	0.0509	161.225	8.298
162	N_M_C2Scap_R_Lev_Scapulae	2008037	0.0299	127.666	22.157
163	N_M_C3Scap_R_Lev_Scapulae	2008038	0.02155	112.242	29.537
164	N_M_C4Scap_R_Lev_Scapulae	2008039	0.0198	101.543	27.078
165	N_M_SC6_R_Longus_Capitis	2008040	0.01115	89.327	1.000
166	N_M_SC5_R_Longus_Capitis	2008041	0.01115	78.348	1.000
167	N_M_SC4_R_Longus_Capitis	2008042	0.01115	66.256	1.000

Continued on next page

Table 1 – Continued from previous page

Nr.	Muscle names	PID	$\mathbf{F_{max}}$ [kN]	$\mathbf{l_{CE,opt}}$ [mm]	$\mathbf{l_{SEE,0}}$ [mm]
168	N_M_SC3_R_Longus_Capitis	2008043	0.01115	51.863	1.000
169	N_M_C4C1_R_Longus_Colli	2008044	0.00615	44.046	1.000
170	N_M_C4C2_R_Longus_Colli	2008045	0.00265	29.202	1.000
171	N_M_C7C5_R_Longus_Colli	2008046	0.00765	41.334	1.000
172	N_M_C7C4_R_Longus_Colli	2008047	0.00765	56.638	1.000
173	N_M_T1C3_R_Longus_Colli	2008048	0.00765	92.116	1.000
174	N_M_T1C2_R_Longus_Colli	2008049	0.00765	104.913	1.000
175	N_M_T1C1_R_Longus_Colli	2008050	0.00765	121.020	1.000
176	N_M_T4C7_R_Multifidus_Cervicis	2008051	0.01745	67.717	9.850
177	N_M_T3C7_R_Multifidus_Cervicis	2008052	0.01945	55.356	1.000
178	N_M_T3C6_R_Multifidus_Cervicis	2008053	0.01945	70.453	1.000
179	N_M_T2C6_R_Multifidus_Cervicis	2008054	0.0347	48.427	1.000
180	N_M_T1C5_R_Multifidus_Cervicis	2008055	0.01895	35.329	1.000
181	N_M_T1C4_R_Multifidus_Cervicis	2008056	0.02915	47.164	1.000
182	N_M_C7C3_R_Multifidus_Cervicis	2008057	0.028	56.346	1.000
183	N_M_C6C3_R_Multifidus_Cervicis	2008058	0.01805	43.066	1.000
184	N_M_C6C2_R_Multifidus_Cervicis	2008059	0.01805	55.815	1.000
185	N_M_C5C2_R_Multifidus_Cervicis	2008060	0.01805	44.573	1.000
186	N_M_C2C1_R_Obliquus_Cap_Inf	2008061	0.08565	45.241	1.000
187	N_M_C1S_R_Obliquus_Cap_Sup	2008062	0.0461	21.878	1.000
188	N_M_ScapH_R_Omohyoid	2008063	0.0222	239.111	1.000
189	N_M_SternH_R_SternoHyoid	2008065	0.0128	141.275	1.000
190	N_M_ClavH_R_SternoHyoid	2008066	0.00425	113.976	1.000
191	N_M_R1H_R_Sternothyroid	2008067	0.00985	130.961	1.000
192	N_M_SternH_R_Sternothyroid	2008068	0.01585	134.756	1.000
193	N_M_C1S_R_Rect_Cap_Ant	2008070	0.004	18.274	1.000
194	N_M_C1S_R_Rect_Cap_Lat	2008071	0.03915	17.222	1.000
195	N_M_C2S_R_Rect_Cap_Post_Maj	2008072	0.02705	42.721	1.000
196	N_M_C1S_R_Rect_Cap_Post_Min	2008073	0.04515	29.635	1.000
197	N_M_C6Scap_R_Rhomb_Min	2008074	0.0239	116.215	1.000
198	N_M_C7Scap_R_Rhomb_Min	2008075	0.0239	109.615	1.000
199	N_M_C4R1_R_Scalenus_Ant	2008076	0.01025	64.683	28.123
200	N_M_C5R1_R_Scalenus_Ant	2008077	0.01025	62.292	17.604
201	N_M_C6R1_R_Scalenus_Ant	2008078	0.02055	57.047	9.921
202	N_M_C1R1_R_Scalenus_Med	2008079	0.01315	65.012	60.678
203	N_M_C2R1_R_Scalenus_Med	2008080	0.01315	65.291	45.704
204	N_M_C3R1_R_Scalenus_Med	2008081	0.01315	73.325	28.108
205	N_M_C4R1_R_Scalenus_Med	2008082	0.01315	66.346	21.010
206	N_M_C5R1_R_Scalenus_Med	2008083	0.01315	59.972	14.993
207	N_M_C6R1_R_Scalenus_Med	2008084	0.01315	50.625	10.969
208	N_M_C7R1_R_Scalenus_Med	2008085	0.01315	49.628	3.309
209	N_M_C5R1_R_Scalenus_Post	2008086	0.0223	62.624	32.392
210	N_M_C6R1_R_Scalenus_Post	2008087	0.0223	61.149	24.249
211	N_M_T4S_R_Semispinalis_Capitis	2008088	0.023	199.272	1.000
212	N_M_T3S_R_Semispinalis_Capitis	2008089	0.0257	175.968	1.000
213	N_M_T2S_R_Semispinalis_Capitis	2008090	0.03145	150.655	1.000
214	N_M_T1S_R_Semispinalis_Capitis	2008091	0.0316	130.571	1.000
215	N_M_C7S_R_Semispinalis_Capitis	2008092	0.02095	120.197	1.000
216	N_M_C6S_R_Semispinalis_Capitis	2008093	0.0172	103.021	1.000
217	N_M_C5S_R_Semispinalis_Capitis	2008094	0.02285	92.347	1.000
218	N_M_C4S_R_Semispinalis_Capitis	2008095	0.02295	80.530	1.000
219	N_M_C3S_R_Semispinalis_Capitis	2008096	0.01765	68.575	1.000
220	N_M_T5C7_R_Semispinalis_Cervicis	2008097	0.0227	117.653	1.000
221	N_M_T5C6_R_Semispinalis_Cervicis	2008098	0.0227	131.975	1.000
222	N_M_T4C5_R_Semispinalis_Cervicis	2008099	0.0136	120.125	1.000
223	N_M_T4C4_R_Semispinalis_Cervicis	2008100	0.0136	131.917	1.000
224	N_M_T4C3_R_Semispinalis_Cervicis	2008101	0.0136	156.857	1.000
225	N_M_T3C4_R_Semispinalis_Cervicis	2008102	0.01765	117.199	1.000
226	N_M_T3C3_R_Semispinalis_Cervicis	2008103	0.01765	137.446	1.000
227	N_M_T3C2_R_Semispinalis_Cervicis	2008104	0.01765	145.575	1.000
228	N_M_T2C3_R_Semispinalis_Cervicis	2008105	0.0225	114.361	1.000

Continued on next page

Table 1 – Continued from previous page

Nr.	Muscle names	PID	$\mathbf{F_{max}}$ [kN]	$\mathbf{l_{CE,opt}}$ [mm]	$\mathbf{l_{SEE,0}}$ [mm]
229	N_M_T2C2_R_Semispinalis_Cervicis	2008106	0.0225	126.073	1.000
230	N_M_T1R4_R_Serratus_Post_Sup	2008107	0.01265	41.544	58.321
231	N_M_C7R4_R_Serratus_Post_Sup	2008108	0.0251	53.975	52.145
232	N_M_C7R3_R_Serratus_Post_Sup	2008109	0.03295	49.302	42.399
233	N_M_C6R1_R_Serratus_Post_Sup	2008110	0.028	24.503	29.403
234	N_M_C5S_R_Splenius_Capitis	2008111	0.0088	87.706	1.000
235	N_M_C6S_R_Splenius_Capitis	2008112	0.01395	103.460	1.000
236	N_M_C6S_R_Splenius_Capitis	2008113	0.0198	105.477	1.000
237	N_M_C7S_R_Splenius_Capitis	2008114	0.01935	121.936	1.000
238	N_M_C7S_R_Splenius_Capitis	2008115	0.01905	124.187	1.000
239	N_M_T1S_R_Splenius_Capitis	2008116	0.01935	140.981	1.000
240	N_M_T2S_R_Splenius_Capitis	2008117	0.0247	176.331	1.000
241	N_M_T3C1_R_Splenius_Cervicis	2008118	0.02475	124.921	56.337
242	N_M_T3C2_R_Splenius_Cervicis	2008119	0.02475	91.638	71.873
243	N_M_SternS_R_SCM1	2008120	0.0349	123.228	58.896
244	N_M_SternS_R_SCM2	2008121	0.062	137.172	41.914
245	N_M_ClavS_R_SCM1	2008122	0.02445	95.452	52.914
246	N_M_ClavS_R_SCM2	2008123	0.0237	115.480	33.489
247	N_M_C5Scap_R_Trap_Desc	2008124	0.05055	115.633	39.420
248	N_M_C5Scap_R_Trap_Desc	2008125	0.07115	128.325	40.955
249	N_M_C5Scap_R_Trap_Desc	2008126	0.0168	193.969	6.520
250	N_M_SClav_R_Trap_Desc	2008127	0.03835	235.309	7.715
251	N_M_C7Scap_R_Trap_Trans	2008128	0.1285	72.874	94.836
252	N_M_C6Scap_R_Trap_Trans	2008129	0.119	66.991	97.910
